# Hyaluronic acid injection therapy for osteoarthritis of the knee: concordant efficacy and conflicting serious adverse events in two systematic reviews

**DOI:** 10.1186/s13643-016-0363-9

**Published:** 2016-11-04

**Authors:** Claire E. O’Hanlon, Sydne J. Newberry, Marika Booth, Sean Grant, Aneesa Motala, Margaret A. Maglione, John D. FitzGerald, Paul G. Shekelle

**Affiliations:** 1 RAND Corporation, 1776 Main St., Santa Monica, CA 90407 USA; 2 Pardee RAND Graduate School, 1776 Main St., Santa Monica, CA 90407 USA; 3 Division of Rheumatology, Department of Internal Medicine, UCLA, 1000 Veteran Avenue, Los Angeles, CA 90095 USA; 4 Veterans Affairs Greater Los Angeles Healthcare System, 11301 Wilshire Boulevard, Los Angeles, CA 90073 USA

**Keywords:** Osteoarthritis, knee, Hyaluronic acid, Viscosupplementation, Adverse events

## Abstract

**Background:**

The prevalence of knee osteoarthritis (OA)/degenerative joint disease (DJD) is increasing in the USA. Systematic reviews of treatment efficacy and adverse events (AEs) of hyaluronic acid (HA) injections report conflicting evidence about the balance of benefits and harms. We review evidence on efficacy and AEs of intraarticular viscosupplementation with HA in older individuals with knee osteoarthritis and account for differences in these conclusions from another systematic review.

**Methods:**

We searched PubMed and eight other databases and gray literature sources from 1990 to December 12, 2014. Double-blind placebo-controlled randomized controlled trials (RCTs) reporting functional outcomes or quality-of-life; RCTs and observational studies on delay/avoidance of arthroplasty; RCTs, case reports, and large cohort studies and case series assessing safety; and systematic reviews reporting on knee pain were considered for inclusion.

A standardized, pre-defined protocol was applied by two independent reviewers to screen titles and abstracts, review full text, and extract details on study design, interventions, outcomes, and quality. We compared our results with those of a prior systematic review and found them to be discrepant; our analysis of why this discrepancy occurred is the focus of this manuscript.

**Results:**

Eighteen RCTs reported functional outcomes: pooled analysis of ten placebo-controlled, blinded trials showed a standardized mean difference of −0.23 (95 % confidence interval (CI) −0.45 to −0.01) favoring HA at 6 months. Studies reported few serious adverse events (SAEs) and no significant differences in non-serious adverse events (NSAEs) (relative risk (RR) [95 % CI] 1.03 [0.93–1.15] or SAEs (RR [95 % CI] 1.39 [0.78–2.47]). A recent prior systematic review reported similar functional outcomes, but significant SAE risk. Differences in SAE inclusion and synthesis accounted for the disparate conclusions.

**Conclusions:**

Trials show a small but significant effect of HA on function on which recent systematic reviews agree, but lack of AE synthesis standardization leads to opposite conclusions about the balance of benefits and harms. A limitation of the re-analysis of the prior systematic review is that it required imputation of missing data.

**Electronic supplementary material:**

The online version of this article (doi:10.1186/s13643-016-0363-9) contains supplementary material, which is available to authorized users.

## Background

Prevalence of osteoarthritis of the knee is increasing rapidly in the USA due to shifting population demographics: primary risk factors include aging, obesity, prior injury, repetitive use, [1], and female gender [2]. The Centers for Disease Control estimate that prevalence of symptomatic knee osteoarthritis may reach 50 % by age 85 [3]. The increase in obesity has translated into not only increasing knee osteoarthritis incidence but also younger age of onset; as a result, by the time individuals reach Medicare eligibility, the length of time they have had the condition has grown, their cases are more advanced [4], and the likelihood of needing surgery has increased.

Traditional treatment options for knee osteoarthritis include both pharmaceutical (analgesics and anti-inflammatory agents) and lifestyle options (physical therapy, exercise, weight loss), as well as surgery (partial or total arthroplasty) for advanced cases. More recent therapies include intraarticular viscosupplementation, which involves local injections of joint lubricant hyaluronic acid (HA) [5].

Recommendations for using HA for knee osteoarthritis have been mixed. In the 2012 update to their 2000 guidelines for the treatment of osteoarthritis of the knee, hip, and hand, the American College of Rheumatology conditionally recommended HA injections for patients who had an inadequate response to initial therapy [5]. The 2013 American Academy of Orthopedic Surgeons guidelines for the treatment of knee osteoarthritis recommend against the use of HA to treat patients with symptomatic conditions [6].

Systematic reviews have an important role in establishing evidence-based clinical guidelines. Much work has been done on improving the methods of systematic reviews for medical treatments, and this work has largely standardized the synthesis of benefits. Clinical and outcomes researchers have created standardized scales and tools to elicit and quantify mean statistical differences. However, clinical guidelines consider the balance of benefits and harms. The elicitation, appraisal, and reporting of harms are far less standardized than for benefits. This lack of standardization can sometimes influence clinical recommendations either for or away from potential treatments.

We report here a comparison of the results of two systematic reviews assessing efficacy and adverse events on the use of HA of patients with knee osteoarthritis. Our starting point is our systematic review and meta-analysis on the use of HA in patients 65 and older commissioned by the U.S. Center for Medicare and Medicaid Services. In the course of comparing our results to a previous systematic review on the same topic, we identified a situation in which differences in how adverse events (AEs) are synthesized have resulted in differences in estimates of the risk of harms, which in turn result in completely different conclusions regarding the balance of benefits and harms for the use of HA, in spite of reporting similar results on effectiveness for functional outcomes. In this paper, we briefly describe the methods and results of our commissioned review (full details are available in our Evidence Report [7]), and then focus on the methods and results of a comparison between the AE results from the two reviews.

## Methods

This systematic review was conducted under contract for the Agency for Healthcare Research and Quality (AHRQ) through its Evidence-based Practice Center (EPC) Program. As the Centers for Medicare and Medicaid Services (CMS) was the partner for this review and the vast majority of Medicare beneficiaries are over 65, the key questions focused on the functional efficacy and safety of intraarticular HA injections for knee osteoarthritis in persons aged 65 years and older. Although the study was not originally registered with PROSPERO, this study followed a pre-defined, standardized protocol approved by the Centers for Medicare and Medicaid Services (CMS) that was posted for public comment. The full report [7] is available at http://www.ncbi.nlm.nih.gov/books/NBK343555/. The PRISMA checklist for this manuscript is available as an additional file.

As part of the interpretation of our findings, we compared our results with those of prior systematic reviews. Discrepancies in the analysis of AEs in RCTs in our review and the analysis of RCTs in Rutjes and colleagues’ review [8] caused us to perform a more detailed analysis of serious adverse events (SAEs) reported in the trials included in both reviews. Our investigation into the causes of such discrepant results is the focus of this manuscript.

### Search strategy and inclusion criteria

The full description of our search strategy is included in our Evidence Report [7]. Briefly, we searched PubMed, EMBASE, Web of Science, Scopus, the Cochrane database, www.clinicaltrials.gov, the Canadian Agency for Drugs and Technologies in Health database, the Food and Drug Administration Premarket Approval database, the New York Academy of Medicine Grey Literature Report, and unpublished documents provided by manufacturers from January 1, 1990, to December 12, 2014. Search strings included a term for the treatment (hyaluronic acid, hyaluronate, hyaluronan, hylan, viscosupplementation, or similar), a term for the disease state (osteoarthritis, arthritis, gonarthrosis, degenerative joint disease), and a term for the site (knee). See Additional file 1: Table S1 for the full search strategy. Non-English language studies and conference abstracts were excluded, although non-USA studies were included if the product evaluated was analogous to a product available in the USA.

We included randomized controlled trials (RCTs) for functional and quality-of-life efficacy outcomes. We included RCTs and cohort studies for total knee replacement (TKR) efficacy outcomes. Recent comprehensive systematic reviews that reported pain outcomes were also included. RCTs, cohort studies, case series, and case studies were included for AE outcomes, although only RCTs contributed to the pooled estimates.

### Screening and data abstraction

Titles and abstracts were independently screened by two reviewers. Data were dually and independently abstracted with disagreements resolved by group discussion. Abstracted data included both study-level data (population demographics, health status, and intervention protocols) and efficacy outcomes of interest. We also abstracted information on AEs.

### Quality assessments

Study quality was assessed using questions adapted from the Cochrane Risk of Bias Assessment Tool [9] and EPC Methods Handbook [10]. The quality of RCTs included in the AE assessment was evaluated using the McHarms tool [11].

### Efficacy and adverse event analyses

Efficacy analyses were conducted with Stata statistical software, version 12.0 (Stata Corp., College Station, TX). Pooling of adverse events was conducted with StatXact PROCS, version 10 (Cytel, Cambridge, MA).

### Efficacy analysis

We conducted meta-analysis of the efficacy outcomes of interest in cases where there were three or more sufficiently homogeneous studies and estimated a pooled random-effects estimate of the overall effect size [12]. We compared these effect sizes to recent estimates of minimum clinically important difference (MCID) for knee osteoarthritis.

### Adverse event analysis

We classified each reported adverse event on two dimensions: severity (either serious [“SAE”] or not serious [“NSAE”]) and locality (local to the injected joint, local but not to the injected joint, or non-local [“other”]). Classifications were determined by board-certified clinicians on the research team, a rheumatologist (J.D.F.) and an internist (P.G.S.). Adverse events were pooled by severity and locality. Pooling of adverse events was conducted using exact methods; events with zeros in one group were included in the analysis while events with zeros in both groups were excluded [13].

### Sensitivity analysis

As part of the interpretation of our findings, we compared our results with those of prior systematic reviews, including a review by Rutjes and colleagues [8], which was the most recent prior review of high quality (quality score assessed by AMSTAR [14]: 9 out of 11) available at the time. While both reviews had concordant efficacy results, our study and their study resulted in different conclusions on the risk of SAEs. We hypothesized that such differences in conclusions could arise from three sources: differences in included studies; differences in AEs included in the studies; and differences in how AEs were classified and synthesized. We investigated each potential source.

### Differences in included serious adverse event studies

We retrieved all studies included in the systematic review by Rutjes and colleagues [8] and compared inclusion criteria and studies included to those of our review. For each study reported in the pooled SAE analysis in the systematic review by Rutjes and colleagues [8], we replicated their pooled analysis to conduct a sensitivity analysis. Because three of the studies included in their meta-analysis were considered proprietary and specific data were withheld from publication, we used the known sample sizes for these three studies and trial-and-error to replicate their pooled result to determine the number of SAEs in these studies. We used this replication to determine how sensitive their conclusions were to inclusion and exclusion of individual studies.

### Differences in reported adverse events

We compiled all NSAEs and SAEs reported in our review and in the review by Rutjes and colleagues [8]. As in our own review, we classified AEs reported by Rutjes and colleagues as serious or non-serious, and then further by whether the AE was local to the knee joint, local to somewhere other than the knee joint, or a non-local AE. Rutjes and colleagues reported results for one NSAE (flare) and then used the original study authors’ assessment of AEs as serious or non-serious for their determination of SAEs. We then compared the types of NSAEs and SAEs reported in our review to the NSAEs and SAEs in the review by Rutjes and colleagues [8].

### Differences in synthesis of adverse events

We compared how Rutjes and colleagues [8] synthesized the evidence on AEs to our methods for synthesizing AEs. We then conducted sensitivity analyses to assess the degree to which modifications to these classifications influence the pooled results.

### Strength of evidence

We assessed the strength of evidence for each outcome using criteria from the Effective Health Care Program [15], which are similar to those used by the Grades of Recommendation Assessment, Development and Evaluation (GRADE) Working Group [16] and include assessments of the study limitations, directness, consistency, precision, and likelihood of reporting bias of the evidence.

### Role of the funding source

The original Evidence Report was funded by AHRQ [7]. No additional funding was obtained for the AE sensitivity analysis work. The results and conclusions are those of the authors, who are solely responsible for deciding to submit this manuscript for publication.

## Results

### Literature flow and efficacy results

**Fig. 1:**
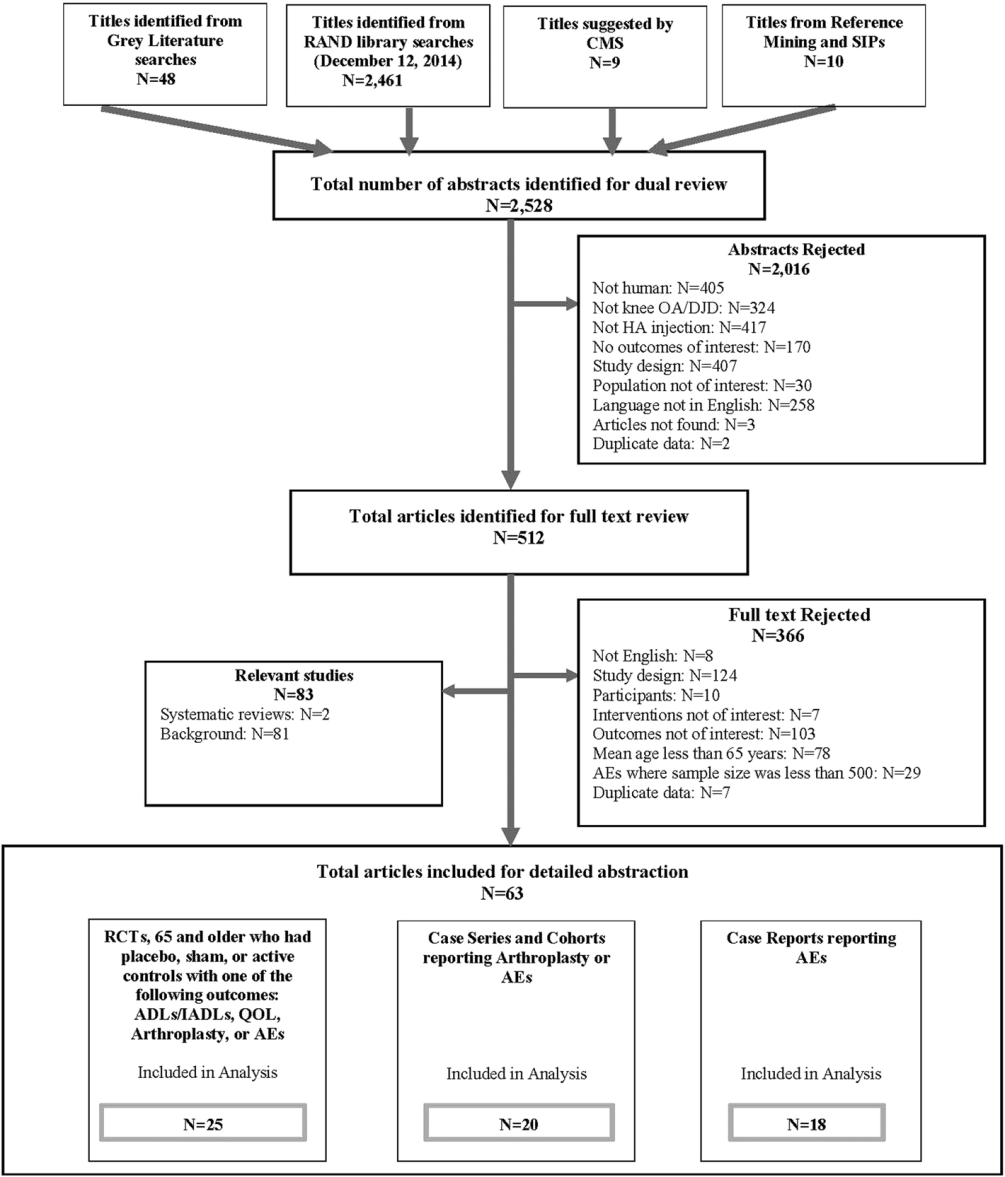
Flow diagram of included articles our systematic review. Of a total of 2528 potential articles, 63 were analyzed in the full report [7]

Of the 2528 articles screened, 512 were selected for review of the full text, and 63 articles met inclusion criteria for our analyses (Fig. 1). Study-level data can be found in the evidence tables (Additional file 2: Table S2).

### Functional efficacy analysis

**Fig. 2:**
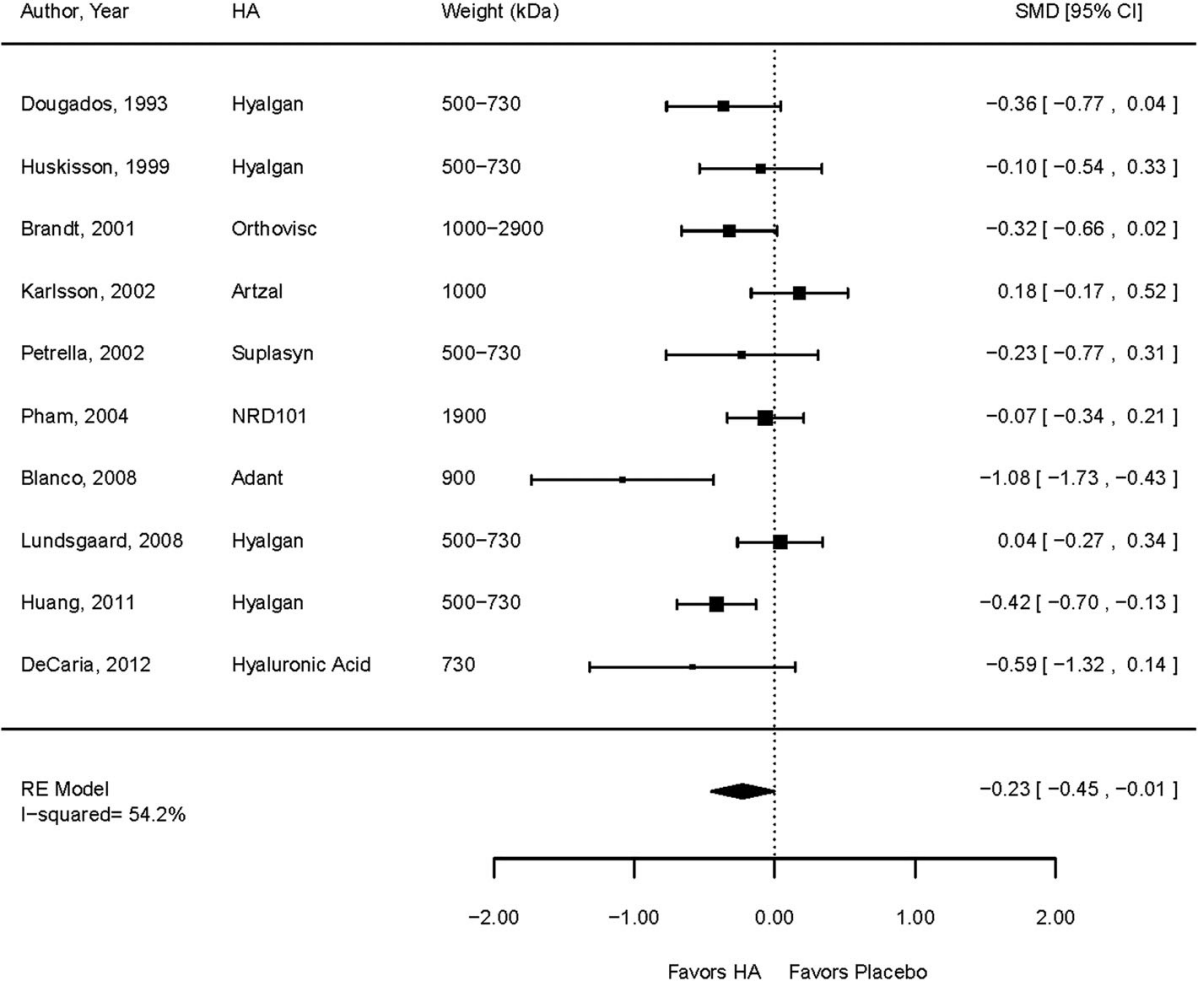
Forest plot for functional efficacy meta-analysis. The effect of hyaluronic acid (HA) injections in knee osteoarthritis on function (as measured by Western Ontario and McMaster Universities Osteoarthritis Index [WOMAC], Lequesne, or Knee Injury and Osteoarthritis Outcomes Score [KOOS] scales) at 26 weeks* follow-up is small but statistically significant. Studies are arranged chronologically. *Follow-up time was 26 weeks for all studies except for Petrella et al. 2002 (4 weeks), Dougados et al. 1993 (52 weeks), and Pham et al. 2004 (52 weeks). *kDa* kilo-Daltons. *SMD* standardized mean difference

The full details of the efficacy analyses are included in our Evidence Report [7]. In brief, 18 randomized trials reported on the effects of HA compared to sham-injected placebo control, another HA, or some other active treatment on function, as measured by the Western Ontario-McMaster Universities Arthritis Index (WOMAC [17]), the Lequesne Index [18], the Knee Injury and Osteoarthritis Outcomes Score (KOOS [19]), or Activities of Daily Living, among patients whose average age was 65 or older. Details of included studies and their risk of bias assessment are included in Additional files 2 and 3. Pooled analysis of ten sham-injection, placebo-controlled, assessor-blinded trials showed a standardized mean difference of −0.23 (95 % CI −0.45 to −0.01) (Fig. 2), which significantly favored HA at 6 months follow-up [20–29]. Although our review found that functional outcomes were improved by intraarticular HA injection, the durability of this effect could not be assessed beyond 6 months. We judged the strength of evidence for the function outcome as low because the trials tended to be small, they had moderate risk of bias (often failing to report adequate methods for recruitment or concealment of allocation) (Additional file 3: Table S3), function was usually not a primary outcome, and results were inconsistent.

Our functional effect size of −0.23 (95 % CI −0.45 to −0.01) is similar to previously reported effect sizes. Rutjes and colleagues report an effect size of −0.33 (95 % CI −0.43 to −0.22) [8], while Bannuru and colleagues report −0.30 (95 % CI −0.40 to −0.20) [30]. Our effect size for function did not exceed the minimum clinically important difference (MCID) of −0.37 applied in the review by Rutjes and colleagues [8] but did exceed the minimum clinically important improvement (MCII) of −0.12 derived by Tubach and colleagues [31], as well as the MCII of −0.20 used by Bannuru and colleagues [30].

### Other efficacy analyses

Quality-of-life outcomes assessed in three RCTs (one placebo-controlled [26] and two head-to-head trials [32, 33]) found no statistically significant differences between groups. Three RCTs [22, 29, 34] and 13 observational studies (reported in 16 articles [35–50]) reported on TKR, but evidence on delay or avoidance of TKR was insufficient to draw conclusions. Two large, good quality systematic reviews with meta-analyses for pain outcomes showed a significant and clinically important effect among adults of all ages [8, 51].

### Adverse event analysis

Twenty-four trials reported data on AEs [20–29, 32, 33, 52–63]. Thirteen trials compared HA to placebo, seven compared HA to an active comparator, and four trials reported data on both comparison types, but only the placebo comparisons [20–29, 33, 53, 54, 56, 60, 62, 63] had enough trials within AE categories to pool. There were few SAEs. We found no significant risk of NSAEs or SAEs compared to placebo overall as well as local, joint, and other NSAEs or SAEs in a stratified analysis (Table 1). However, the 95 % confidence intervals were wide; a clinically important effect could not be excluded. Study quality was assessed using the McHarms tool [11], which is described in depth in the Evidence Report) [7]. We judged the strength of evidence as Moderate and Low that there is no significantly increased risk between HA and placebo in the rate of NSAEs and SAEs, respectively.

**Table 1:** Adverse event analysis

AE category	Number of studies	Number of events, HA group	Sample size, HA group	Number of events, placebo group	Sample size, placebo group	Relative risk	95 % confidence interval
All serious AEs (combined joint and other^a^)	*8*	*27*	*1056*	*16*	*1017*	*1.39*	(*0.78, 2.47*)
Serious AEs, joint (e.g., synovitis)	5	10	442	8	447	1.25	(0.53, 2.94)
Serious AEs, other (e.g., herpes zoster)	6	17	614	8	570	1.52	(0.70, 3.26)
All non-serious AEs (combined joint, local non-joint, and other)	*10*	*415*	*1645*	*375*	*1564*	*1.03*	(*0.93, 1.15*)
Non-serious AEs, joint (e.g., pain)	7	121	559	97	518	1.15	(0.91, 1.43)
Non-serious AEs, local non-joint (e.g., erythema)	6	98	492	79	493	1.26	(0.99, 1.60)
Non-serious AEs, other (e.g., headache)	6	196	594	199	553	0.89	(0.78, 1.03)

Our review finds no significant risk of adverse events (NSAEs) or serious adverse events (SAEs) associated with hyaluronic acid (HA) injections in knee osteoarthritis (confidence intervals cross 1; italicized rows). Subgroup analysis by AE category (joint, other, local non-joint^a^) also found no significant risk of NSAEs or SAEs (confidence intervals cross 1; plain text rows)

^a^No studies reported local non-joint SAEs

### Sensitivity analysis

While neither our study nor the study by Rutjes and colleagues found a significantly increased risk of NSAEs, the two studies reached different conclusions about the risk of SAEs. Our review concluded that the use of HA injections showed no evidence of a statistically significant increase in local, joint, or other SAEs: 1.39 (95 % CI 0.78–2.47) (Table 2). Rutjes and colleagues [8] did report a statistically significant relative risk of SAEs: 1.41 (95 % CI 1.02–1.97) for all studies and 1.55 (95 % CI 1.07–2.24) for a subgroup of only “large, blinded studies” (Table 2). This result was instrumental in their conclusion that, “In patients with knee osteoarthritis, viscosupplementation is associated with a small and clinically irrelevant benefit and an increased risk for serious adverse events.”

**Table 2:** Sensitivity analysis of the risk of serious adverse events associated with hyaluronic acid for osteoarthritis of the knee

	Relative risk (RR) (95 % confidence interval (CI))	RR (95 % CI) excluding outlier study by Jubb et al. [64]
Pooled SAE results in our review	1.39 (0.78–2.47)	–
Original pooled SAE results in Rutjes et al. [8]		
- All studies	1.41 (1.02–1.97)	–
- Sample restricted to “large, blinded trials”	1.55 (1.07–2.24)	–
Our approximated pooled SAE results (data imputed for studies in Rutjes et al. [8] with proprietary data)		
- All studies	1.42 (1.01–1.99)	1.26 (0.83–1.90)
- Sample restricted to “large, blinded trials”	1.54 (1.05–2.28)	1.37 (0.83–2.26)
Approximated pooled SAE results including omitted SAEs (two cases of myocardial infarction [54, 75], four cases of severe knee swelling [54], one case of cerebral hemorrhage [26], and one case of breast cancer [75])		
- All studies	1.37 (0.98–1.91)	1.21 (0.82–1.80)
- Sample restricted to “large, blinded trials”	1.50 (1.03–2.19)	1.32 (0.82–2.13)
Approximated pooled SAE results excluding four cancer cases [73]		
- All studies	1.36 (0.97–1.89)	1.20 (0.81–1.77)
- Sample restricted to “large, blinded trials”	1.48 (1.02–2.16)	1.30 (0.81–2.09)
Approximated pooled SAE results including omitted non-cancer SAEs (two cases of myocardial infarction [54, 75], four cases of severe knee swelling [54], one case of cerebral hemorrhage [26]) and excluding four cancer cases [73]		
- All studies	1.37 (0.98–1.91)	1.21 (0.81–1.80)
- Sample restricted to “large, blinded trials”	1.50 (1.03–2.18)	1.32 (0.82–2.12)

This table compares the SAE results in our review, the review by Rutjes et al. [8], our approximation of their results, and the sensitivity of those approximated results to the inclusions and exclusion of individual AEs and the outlier study by Jubb et al. [64]

### Differences in included adverse event studies

Our review and the review by Rutjes and colleagues included only two of the same studies in the analyses of SAEs (Additional file 4: Table S4) [23, 24]. At least some, but not all, of this difference is attributable to different study inclusion criteria. While both SAE analyses considered only randomized, placebo-controlled studies, we applied somewhat stricter criteria to the allowable study designs (e.g., excluding studies where a patient’s contralateral knee served as “control”). Although in our full report we summarize AEs garnered from case reports and large observational studies [7], results from studies with these designs were not pooled and are not discussed here. However, the most important difference in inclusion criteria between these two reviews was that our review was restricted to studies where the average age was 65 or older, to match the population of interest for CMS. Studies considered for inclusion in the analysis by Rutjes and colleagues were not restricted by age.

We then attempted to re-evaluate the sensitivity of Rutjes’ results to the inclusion and exclusion of individual studies. We were able to calculate a close approximation of their pooled results by imputing data for the three studies with missing proprietary data. Our approximation of their results was 1.42 (95 % CI 1.01–1.99) (compared to their result of 1.41 [95 % CI 1.02–1.97]) and 1.54 (95 % CI 1.05–2.28) for “large, blinded trials” (compared to their result of 1.55 [95 % CI 1.07–2.24]). Using these imputed results, we determined that the pooled results are sensitive to the inclusion or exclusion of a single study by Jubb and colleagues [64]—it has a weight in the pooled analysis of 31 %. This study is an outlier compared to the other studies with respect to the rate of SAEs reported, with most studies reporting SAE rates of 2–3 %, while Jubb and colleagues report a rate three times higher (7 %). We note that excluding this study from the analysis removes the significance of the finding by Rutjes and colleagues, yielding relative risks of 1.26 (95 % CI 0.83–1.90) for all studies and 1.37 (95 % CI 0.83–2.26) in “large, blinded studies” (Table 2), results similar to our pooled SAE results. The sensitivity of the result by Rutjes and colleagues to a single outlier study prompted us to investigate what the SAEs in this study actually were.

### Differences in reported adverse events

Jubb and colleagues report that they questioned patients about AEs, which is known to increase the rates at which AEs are reported [65, 66]; however, they did not report what the 14 SAEs in the placebo group and the 27 in the treatment group were (except for one death in the treatment group), only that they were “serious”. In addition, they declare that “all serious AEs were considered by the investigators to be the result of primary concomitant disease and not to be drug-related.” Since we could not further analyze this claim, we investigated the SAEs reported in the other studies analyzed by Rutjes and colleagues.

Of the 14 studies analyzed by Rutjes and colleagues, only three included what constituted an SAE for each treatment group (Additional file 5: Table S5) [23, 24, 67]. An additional three were the sources of the previously mentioned unreported proprietary data; four presented SAEs without additional specification of what qualified as an SAE [64, 68–70]; two presented what they included as SAEs in aggregate but not by treatment group [71, 72]; one reported a mix of specific and nonspecific SAEs [73]; and for one, we were unable to find the original article because the journal is out of print and the authors did not respond to our queries [74].

We then compiled a list of NSAEs and SAEs reported in each review, using that review’s classification scheme (Additional file 6: Table S6). This table identifies differences between the reviews in how AEs were categorized and grouped for analysis, especially which AEs were considered serious or non-serious. For example, “joint sprain” was considered an SAE in the review by Rutjes and colleagues whereas it was considered an NSAE in our review; “cancer” was considered an SAE by Rutjes and colleagues whereas it was not considered an AE of any kind in our review.

### Differences in synthesis of adverse events

A fundamental difference in the two methods for considering AEs was the use of clinical judgment by the systematic review team. We used clinical judgment of experts on our team to decide whether an AE was serious or non-serious or even an AE at all (e.g., cancer), while Rutjes and colleagues adopted the designation of serious or non-serious used by the clinical judgment of the original study authors and considered anything listed as an AE as possibly causally related, even if the study investigators considered the SAE unrelated to the treatment (which was the case for all SAEs in 9 of 10 studies available). The adoption of the study authors’ designation of seriousness results in the omission of AEs from the Rutjes and colleagues SAE pooling that seem qualitatively similar to SAEs they do count. Instances of cases where AEs were excluded because the author did not designate it as serious includes deaths from myocardial infarction in both intervention [54] and control [75] patients, four cases of “severe” knee swelling [54], one case of cerebral hemorrhage in the control group [26], and one case of breast cancer in the control group [75]. This adoption of the author’s designation of seriousness also results in the inclusion of four cancers as relevant SAEs in the course of a 13-week study [73].

To estimate the sensitivity of these decisions, we removed these four cancer cases from the meta-analysis while retaining all other SAEs; doing so makes the pooled estimate no longer statistically significant (1.36 [95 % CI 0.97–1.89]) (Table 2). Redoing the analysis including the above qualitatively similar AEs that were omitted (two cases of myocardial infarction, four cases of severe knee swelling, and one case of breast cancer) because study authors did not designate them as serious yields a pooled relative risk of 1.37 (95 % CI 0.98–1.91) for all studies and 1.50 (95 % CI 1.03–2.19) for “large, blinded studies” (Table 2). Results that exclude the cancers but include the non-cancer omitted AEs yield a pooled relative risk of 1.37 (95 % CI 0.98–1.91) for all studies and 1.50 (95 % CI 1.03–2.18) for “large, blinded studies,” even when the study by Jubb and colleagues is retained (Table 2). If we consider this study as an outlier (as noted above, it does not present detail about what is considered an SAE and reports extremely high SAEs compared to other studies) and remove it from the analysis, no pooled results are statistically significant.

## Discussion

Our principal finding is that lack of agreed-upon standards for AE reporting and synthesis resulted in two rationally conducted, peer-reviewed meta-analyses that agree on the benefits of an intervention but disagree on the risk of harms, ultimately leading to completely opposite conclusions about the balance of benefits and harms. Our systematic review found that HA injections for knee osteoarthritis provide a small, statistically significant benefit to patients with respect to function, but the clinical significance of this average effect depends on the threshold used. This is broadly consistent with the analysis by Rutjes and colleagues. However, our meta-analysis and that of Rutjes and colleagues differed in their conclusions about the risk of SAEs; this difference is primarily attributable to differences in the way the two reviews considered the AEs trial authors reported, and how they were pooled. Our review abstracted all AEs and then used clinical judgment to categorize them as serious or non-serious, rejected some AEs as physiologically implausible (e.g., cancer diagnosis during a 13-week clinical trial), but we permitted others (e.g., myocardial infarction, intestinal obstruction, gastrointestinal bleeding) where causal plausibility was low, but not physiologically or temporally impossible. Rutjes and colleagues considered any AE designated “serious” by the original authors to be an SAE.

Both strategies have strengths and weaknesses. Certainly, examples of AEs acting through unexpected pathways that are first identified as causal by aggregating clinical trial results exist; disregarding AEs as implausible risks missing these signals. In general, our approach agreed with this. However, when the known biology of a condition precludes a causal pathway, the clinical face validity of this review would be compromised if we included a cancer diagnosis as a causally related AE of HA. Accepting the original study authors’ designation of seriousness also results in inconsistency regarding what counts as an SAE between studies (e.g., inconsistent designation of myocardial infarction and cancer as SAEs), which is then carried over into pooled analyses.

A second difference between the two reviews was in the conduct of sensitivity analyses. Our approximation of the SAE analysis by Rutjes and colleagues found that their statistically significant pooled result is sensitive to the inclusion of a single study [64], which carries nearly a third of the weight in the analysis. Although this study was judged as moderate quality on the McHarms tool, it provided few details and reported conspicuously high rates of SAEs compared to other studies. Furthermore, the authors of that study concluded that SAEs reported were attributable to concomitant disease. Combined, this suggests that it is premature to conclude that HA “is associated with an increased risk for serious adverse events.” However, we cannot rule out the possibility of an increased risk of SAE from HA, as the number of studies that have assessed AEs relative to the total number of studies is small and they have lacked methodological rigor. This is a common problem with AE assessments in RCTs and our 95 % CIs for AEs were wide. We also could not conclude there is evidence of a significantly increased risk of SAEs in this treatment. Whether or not the risk of SAEs is significant is critical to clinical decision-making about HA. Since systematic reviews agree HA has a clinically modest benefit on average, if a review concludes there is evidence of a statistically significantly increased risk of SAEs, harms may outweigh benefits and HA should not be offered (as Rutjes and colleagues conclude). But if a review concludes there is no such evidence, then benefits may outweigh harms and HA may be offered (as we conclude).

This study had several limitations. In our CMS-supported review, we did not attempt to review studies of populations with average age below 65, since our study protocol was aimed at producing evidence for the Medicare population. We did not include non-English studies or conference abstracts. Our sensitivity analyses relied on an approximate replication of the meta-analysis by Rutjes and colleagues, though our approximation is very close to their results. Lastly, our searches ended in 2014, although updating the searches would not impact on the comparison of SAE results between our 2015 review and other contemporaneous reviews, which is the main subject of this paper.

## Conclusions

We have identified a situation in which the lack of standardization for AE reporting and synthesis leads to in totally different conclusions about whether the risk of SAEs for a common treatment outweighs its likely benefits. We are not the first to observe the need to standardize the elicitation, reporting, and evaluation of harms in the assessment of medical treatments [76]. A step in the right direction may be the ACTTION (Analgesic, Anesthetic, and Addiction Clinical Trial Translations, Innovations, Opportunities, and Networks) AE checklist to improve the accuracy and completeness of AE data abstracted from reports of trials [77]. However, it is not enough to improve harm elicitation and reporting; the systematic review community also must standardize approaches to AE analysis. Clinicians and policymakers rely on systematic review experts to provide the best available evidence synthesis and they will rightly wonder how they can rely on systematic reviews to evaluate the relative benefits and harms of treatments when faced with situations like this. The systematic review community must remedy the situation.

## Additional files

Additional file 1: Table S1. Search methodology. (DOC 29 kb)
Additional file 2: Table S2. Evidence table. (DOCX 91 kb)
Additional file 3: Table S3. Risk of bias assessment. (DOCX 958 kb)
Additional file 4: Table S4. Comparison of articles contributing to AE and SAE analyses in our review and the review by Rutjes and colleagues [8]. (DOCX 33 kb)
Additional file 5: Table S5. Definitions and counts of SAEs in the review by Rutjes and colleagues [8]. (DOCX 45 kb)
Additional file 6: Table S6. Comparison of AE and SAE categorization in our review and the review by Rutjes and colleagues [8]. Both articles categorized AEs as serious or non-serious. Our review also categorized by locality (joint, non-joint local, or other). Both reviews omitted some SAEs that were qualitatively similar to SAEs counted by the other group. Our review excluded events only that did not describe the type of SAE that occurred. Rutjes et al. omitted some events that were similar to events they did count as SAEs. (DOCX 38 kb)

## Abbreviations

AE: Adverse event(s)
AHRQ: Agency for Healthcare Research and Quality
CMS: Centers for Medicare and Medicaid Services
DJD: Degenerative joint disease
EPC: Evidence-based Practice Center
HA: Hyaluronic acid
KOOS: Knee injury and osteoarthritis outcomes score
MCID: Minimum clinically important difference
OA: Osteoarthritis
RCT(s): Randomized controlled trial(s)
SAE: Serious adverse event
TKR: Total knee replacement
WOMAC: Western Ontario-McMaster Universities Arthritis Index

## References

[1] Richmond SA, Fukuchi RK, Ezzat A, Schneider K, Schneider G, Emery CA (2013). Are joint injury, sport activity, physical activity, obesity, or occupational activities predictors for osteoarthritis? a systematic review. J Orthop Sports Phys Ther.

[2] Losina E, Weinstein AM, Reichmann WM, Burbine SA, Solomon DH, Daigle ME, Rome BN, Chen SP, Hunter DJ, Suter LG (2013). Lifetime risk and age at diagnosis of symptomatic knee osteoarthritis in the US. Arthritis Care Res.

[3] Murphy L, Schwartz TA, Helmick CG, Renner JB, Tudor G, Koch G, Dragomir A, Kalsbeek WD, Luta G, Jordan JM (2008). Lifetime risk of symptomatic knee osteoarthritis. Arthritis Rheum.

[4] Teichtahl AJ, Wluka AE, Wang Y, Strauss BJ, Proietto J, Dixon JB, Jones G, Forbes A, Kouloyan-Ilic S, Martel-Pelletier J (2013). The longitudinal relationship between changes in body weight and changes in medial tibial cartilage, and pain among community-based adults with and without meniscal tears. Ann Rheum Dis.

[5] Hochberg MC, Altman RD, April KT, Benkhalti M, Guyatt G, McGowan J, Towheed T, Welch V, Wells G, Tugwell P (2012). American College of Rheumatology 2012 recommendations for the use of nonpharmacologic and pharmacologic therapies in osteoarthritis of the hand, hip, and knee. Arthritis Care Res (Hoboken).

[6] Treatment of Osteoarthritis of the Knee, 2nd Edition: SUMMARY OF RECOMMENDATIONS [http://www.aaos.org/research/guidelines/OAKSummaryofRecommendations.pdf]. Accessed 20 Feb 2015.

[7] Newberry SJ, Fitzgerald JD, Maglione MA, O'Hanlon CE, Booth M, Motala A, Timmer M, Shanman R, Shekelle PG (2015). AHRQ technology assessments. Systematic review for effectiveness of hyaluronic acid in the treatment of severe degenerative joint disease (DJD) of the knee.

[8] Rutjes AW, Juni P, da Costa BR, Trelle S, Nuesch E, Reichenbach S (2012). Viscosupplementation for osteoarthritis of the knee: a systematic review and meta-analysis. Ann Intern Med.

[9] Higgins JP, Altman DG, Gotzsche PC, Juni P, Moher D, Oxman AD, Savovic J, Schulz KF, Weeks L, Sterne JA (2011). The Cochrane Collaboration’s tool for assessing risk of bias in randomised trials. BMJ.

[10] Viswanathan M, Ansari MT, Berkman ND, Chang S, Hartling L, McPheeters M, Santaguida PL, Shamliyan T, Singh K, Tsertsvadze A (2008). Assessing the risk of bias of individual studies in systematic reviews of health care interventions.

[11] Chou R, Aronson N, Atkins D, et al. Assessing harms when comparing medical interventions. In: Agency for Healthcare Research and Quality. Methods Reference Guide for Comparative Effectiveness Reviews [posted November 2008]. Rockville, MD. Available at: http://effectivehealthcare.ahrq.gov/healthInfo.cfm?infotype=rr&ProcessID=60. 21433406

[12] Cornell JE, Mulrow CD, Localio R, Stack CB, Meibohm AR, Guallar E, Goodman SN (2014). Random-Effects Meta-analysis of Inconsistent Effects: A Time for Change. Ann Intern Med.

[13] Mehta CR, Patel NR, Gray R (1985). Computing an exact confidence interval for the common odds ratio in several 2× 2 contingency tables. J Am Stat Assoc.

[14] Shea BJ, Grimshaw JM, Wells GA, Boers M, Andersson N, Hamel C, Porter AC, Tugwell P, Moher D, Bouter LM (2007). Development of AMSTAR: a measurement tool to assess the methodological quality of systematic reviews. BMC Med Res Methodol.

[15] Methods Guide for Effectiveness and Comparative Effectiveness Reviews. AHRQ Publication No. 10(14)-EHC063-EF. Rockville: Agency for Healthcare Research and Quality; 2014. Chapters available at: www.effectivehealthcare.ahrq.gov. 21433403

[16] Michael CW, Stanley Ip, Melissa M, Tim SC, Roger C, Kathleen NL, Karen R, Kathryn M, Evelyn W. Chapter 15: Grading the Strength of a Body of Evidence When Assessing Health Care Interventions for the Effective Health Care Program of the Agency for Healthcare Research and Quality: An Update. In: Methods Guide for Effectiveness and Comparative Effectiveness Reviews. AHRQ Publication No. 10(14)-EHC063-EF. Rockville: Agency for Healthcare Research and Quality; 2014.

[17] Bellamy N, Buchanan WW, Goldsmith CH, Campbell J, Stitt LW (1988). Validation study of WOMAC: a health status instrument for measuring clinically important patient relevant outcomes to antirheumatic drug therapy in patients with osteoarthritis of the hip or knee. J Rheumatol.

[18] Lequesne MG, Mery C, Samson M, Gerard P (1987). Indexes of severity for osteoarthritis of the hip and knee. Validation—value in comparison with other assessment tests. Scand J Rheumatol Suppl.

[19] Roos EM, Roos HP, Lohmander LS, Ekdahl C, Beynnon BD (1998). Knee Injury and Osteoarthritis Outcome Score (KOOS)—development of a self-administered outcome measure. J Orthop Sports Phys Ther.

[20] Brandt KD, Block JA, Michalski JP, Moreland LW, Caldwell JR, Lavin PT (2001). Efficacy and safety of intraarticular sodium hyaluronate in knee osteoarthritis. ORTHOVISC Study Group. Clin Orthop Relat Res.

[21] DeCaria JE, Montero-Odasso M, Wolfe D, Chesworth BM, Petrella RJ (2012). The effect of intra-articular hyaluronic acid treatment on gait velocity in older knee osteoarthritis patients: a randomized, controlled study. Arch Gerontol Geriatr.

[22] Dougados M, Nguyen M, Listrat V, Amor B (1993). High molecular weight sodium hyaluronate (hyalectin) in osteoarthritis of the knee: a 1 year placebo-controlled trial. Osteoarthritis Cartilage.

[23] Huang TL, Chang CC, Lee CH, Chen SC, Lai CH, Tsai CL (2011). Intra-articular injections of sodium hyaluronate (Hyalgan(R)) in osteoarthritis of the knee. a randomized, controlled, double-blind, multicenter trial in the Asian population. BMC Musculoskelet Disord.

[24] Huskisson EC, Donnelly S (1999). Hyaluronic acid in the treatment of osteoarthritis of the knee. Rheumatology (Oxford).

[25] Karlsson J, Sjogren LS, Lohmander LS (2002). Comparison of two hyaluronan drugs and placebo in patients with knee osteoarthritis. A controlled, randomized, double-blind, parallel-design multicentre study. Rheumatology (Oxford).

[26] Lundsgaard C, Dufour N, Fallentin E, Winke P, Gluud C (2008). Intra-articular sodium hyaluronate 2 mL versus physiological saline 20 mL versus physiological saline 2 mL for painful knee osteoarthritis: a randomized clinical trial. Scand J Rheumatol.

[27] Petrella RJ, DiSilvestro MD, Hildebrand C (2002). Effects of hyaluronate sodium on pain and physical functioning in osteoarthritis of the knee: a randomized, double-blind, placebo-controlled clinical trial. Arch Intern Med.

[28] Pham T, Le Henanff A, Ravaud P, Dieppe P, Paolozzi L, Dougados M (2004). Evaluation of the symptomatic and structural efficacy of a new hyaluronic acid compound, NRD101, in comparison with diacerein and placebo in a 1 year randomised controlled study in symptomatic knee osteoarthritis. Ann Rheum Dis.

[29] Blanco FJ, Fernandez-Sueiro JL, Pinto-Tasende JA, Fernandez Lopez JC, Ramallal M, Freire A, Galdo F (2008). Intra-articular hyaluronan treatment of patients with knee osteoarthritis waiting for replacement surgery. Open Arthritis J.

[30] Bannuru RR, Schmid CH, Kent DM, Vaysbrot EE, Wong JB, McAlindon TE (2015). Comparative effectiveness of pharmacologic interventions for knee osteoarthritis: a systematic review and network meta-analysis. Ann Intern Med.

[31] Tubach F, Ravaud P, Martin-Mola E, Awada H, Bellamy N, Bombardier C, Felson DT, Hajjaj-Hassouni N, Hochberg M, Logeart I (2012). Minimum clinically important improvement and patient acceptable symptom state in pain and function in rheumatoid arthritis, ankylosing spondylitis, chronic back pain, hand osteoarthritis, and hip and knee osteoarthritis: results from a prospective multinational study. Arthritis Care Res (Hoboken).

[32] Khanasuk Y, Dechmaneenin T, Tanavalee A (2012). Prospective randomized trial comparing the efficacy of single 6-ml injection of hylan G-F 20 and hyaluronic acid for primary knee arthritis: a preliminary study. J Med Assoc Thai.

[33] Raman R, Dutta A, Day N, Sharma HK, Shaw CJ, Johnson GV (2008). Efficacy of Hylan G-F 20 and Sodium Hyaluronate in the treatment of osteoarthritis of the knee—a prospective randomized clinical trial. Knee.

[34] Forster MC, Straw R (2003). A prospective randomised trial comparing intra-articular Hyalgan injection and arthroscopic washout for knee osteoarthritis. Knee.

[35] Barrett JP, Siviero P (2002). Retrospective study of outcomes in hyalgan(R)-treated patients with osteoarthritis of the knee. Clin Drug Investig.

[36] Campbell DG, Angel KR, Dobson PJ, Lewis PL, Tandon S (2004). Experiences of viscosupplementation for knee osteoarthritis. Aust Fam Physician.

[37] Evanich JD, Evanich CJ, Wright MB, Rydlewicz JA (2001). Efficacy of intraarticular hyaluronic acid injections in knee osteoarthritis. Clin Orthop Relat Res.

[38] Mazieres B, Bard H, Ligier M, Bru I, d'Orsay GG, Le Pen C (2007). Medicoeconomic evaluation of hyaluronic acid for knee osteoarthritis in everyday practice: the MESSAGE study. Joint Bone Spine.

[39] Waddell DD, Joseph B. Delayed total knee replacement with Hylan G-F 20. J Knee Surg. 2014. 10.1055/s-0034-1395281 25349988

[40] Neustadt DH (2003). Long-term efficacy and safety of intra-articular sodium hyaluronate (Hyalgan) in patients with osteoarthritis of the knee. Clin Exp Rheumatol.

[41] Romero Jurado M, Enrique Fidalgo A, Rodriguez Villar V, Mar Medina J, Soler Lopez B (2013). Factors related with the time to surgery in waiting-list patients for knee prostheses. Reumatologia Clinica.

[42] Turajane T, Amphansap T, Labpiboonpong V, Maungsiri S (2009). Total knee replacement following repeated cycles of intra-articular sodium hyaluronate (500-730 Kda) in failed conservative treatment of knee osteoarthritis: a 54-month follow-up. J Med Assoc Thai.

[43] Turajane T, Labpiboonpong V, Maungsiri S (2007). Cost analysis of intra-articular sodium hyaluronate treatment in knee osteoarthritis patients who failed conservative treatment. J Med Assoc Thai.

[44] Turajane T, Tanavaree A, Labpiboonpong V, Maungsiri S (2007). Outcomes of intra-articular injection of sodium hyaluronate for the treatment of osteoarthritis of the knee. J Med Assoc Thai.

[45] Waddell DD, Bricker DC (2006). Clinical experience with the effectiveness and tolerability of hylan G-F 20 in 1047 patients with osteoarthritis of the knee. J Knee Surg.

[46] Waddell DD, Cefalu CA, Bricker DC (2005). A second course of hylan G-F 20 for the treatment of osteoarthritic knee pain: 12-month patient follow-up. J Knee Surg.

[47] Anand A, Balduini F, Rogers K (2010). Hyaluronic acid in management of advanced osteoarthritis of the knee: retrospective analysis. Eur J Orthop Surg Traumatol.

[48] Korkmaz M, Erdogan Y, Okur A, Gocmen AY, Gunaydin I (2013). Comparison of the effects of intraarticular hyaluronic acid and antiinflammatory drug treatments on the surgical intervention rates in patients with gonarthrosis. Turkish J Med Sci.

[49] Whitman CS, Allen D, Comadoll JL, Thomason HC, Oweida SJ (2010). A retrospective study of SUPARTZ and repeat treatment for osteoarthritis pain in the knee. J Manag Care Med.

[50] Waddell DD, Bricker DC (2007). Total knee replacement delayed with Hylan G-F 20 use in patients with grade IV osteoarthritis. J Manag Care Pharm.

[51] Colen S, van den Bekerom MP, Mulier M, Haverkamp D (2012). Hyaluronic acid in the treatment of knee osteoarthritis: a systematic review and meta-analysis with emphasis on the efficacy of different products. BioDrugs.

[52] Dixon AS, Jacoby RK, Berry H, Hamilton EB (1988). Clinical trial of intra-articular injection of sodium hyaluronate in patients with osteoarthritis of the knee. Curr Med Res Opin.

[53] Grecomoro G, Martorana U, Di Marco C (1987). Intra-articular treatment with sodium hyaluronate in gonarthrosis: a controlled clinical trial versus placebo. Pharmatherapeutica.

[54] Altman RD, Moskowitz R (1998). Intraarticular sodium hyaluronate (Hyalgan) in the treatment of patients with osteoarthritis of the knee: a randomized clinical trial. Hyalgan Study Group. J Rheumatol.

[55] Berenbaum F, Grifka J, Cazzaniga S, D'Amato M, Giacovelli G, Chevalier X, Rannou F, Rovati LC, Maheu E (2012). A randomised, double-blind, controlled trial comparing two intra-articular hyaluronic acid preparations differing by their molecular weight in symptomatic knee osteoarthritis. Ann Rheum Dis.

[56] Henderson EB, Smith EC, Pegley F, Blake DR (1994). Intra-articular injections of 750 kD hyaluronan in the treatment of osteoarthritis: a randomised single centre double-blind placebo-controlled trial of 91 patients demonstrating lack of efficacy. Ann Rheum Dis.

[57] Kahan A, Lleu PL, Salin L (2003). Prospective randomized study comparing the medicoeconomic benefits of Hylan GF-20 vs. conventional treatment in knee osteoarthritis. Joint Bone Spine.

[58] Leopold SS, Redd BB, Warme WJ, Wehrle PA, Pettis PD, Shott S (2003). Corticosteroid compared with hyaluronic acid injections for the treatment of osteoarthritis of the knee. A prospective, randomized trial. J Bone Joint Surg Am.

[59] Pavelka K, Uebelhart D (2011). Efficacy evaluation of highly purified intra-articular hyaluronic acid (Sinovial((R))) vs hylan G-F20 (Synvisc((R))) in the treatment of symptomatic knee osteoarthritis. A double-blind, controlled, randomized, parallel-group non-inferiority study. Osteoarthritis Cartilage.

[60] Petrella RJ, Cogliano A, Decaria J (2008). Combining two hyaluronic acids in osteoarthritis of the knee: a randomized, double-blind, placebo-controlled trial. Clin Rheumatol.

[61] Roman JA, Chismol J, Morales M, Donderis JL (2000). Intra-articular treatment with hyaluronic acid. Comparative study of Hyalgan and Adant. Clin Rheumatol.

[62] Tamir E, Robinson D, Koren R, Agar G, Halperin N (2001). Intra-articular hyaluronan injections for the treatment of osteoarthritis of the knee: a randomized, double blind, placebo controlled study. Clin Exp Rheumatol.

[63] Petrella RJ, Decaria J, Petrella MJ (2011). Long term efficacy and safety of a combined low and high molecular weight hyaluronic acid in the treatment of osteoarthritis of the knee. Rheumatol Rep.

[64] Jubb RW, Piva S, Beinat L, Dacre J, Gishen P (2003). A one-year, randomised, placebo (saline) controlled clinical trial of 500-730 kDa sodium hyaluronate (Hyalgan) on the radiological change in osteoarthritis of the knee. Int J Clin Pract.

[65] Ioannidis JPA, Mulrow CD, Goodman SN (2006). Adverse events: the more you search, the more you find. Ann Intern Med.

[66] Bent S, Padula A, Avins AL (2006). Brief communication: better ways to question patients about adverse medical events: a randomized, controlled trial. Ann Intern Med.

[67] Raynauld JP, Torrance GW, Band PA, Goldsmith CH, Tugwell P, Walker V, Schultz M, Bellamy N (2002). A prospective, randomized, pragmatic, health outcomes trial evaluating the incorporation of hylan G-F 20 into the treatment paradigm for patients with knee osteoarthritis (Part 1 of 2): clinical results. Osteoarthritis Cartilage.

[68] Altman RD, Akermark C, Beaulieu AD, Schnitzer T (2004). Efficacy and safety of a single intra-articular injection of non-animal stabilized hyaluronic acid (NASHA) in patients with osteoarthritis of the knee. Osteoarthritis Cartilage.

[69] Blanco F, Fernández-Sueiro J, Pinto-Tasende J, Fernández-López J, Ramallal M, Freire A, Galdo F (2008). Intra-articular hyaluronan treatment of patients with knee osteoarthritis waiting for replacement surgery. Open Arthritis J.

[70] Sanofi-Aventis (2008). A double blind, randomized trial of intra-articular injections of 20 mg of HYALGAN® for the treatment of knee pain due to osteoarthritis.

[71] Neustadt D, Caldwell J, Bell M, Wade J, Gimbel J (2005). Clinical effects of intraarticular injection of high molecular weight hyaluronan (Orthovisc) in osteoarthritis of the knee: a randomized, controlled, multicenter trial. J Rheumatol.

[72] Altman RD, Rosen JE, Bloch DA, Hatoum HT, Korner P (2009). A double-blind, randomized, saline-controlled study of the efficacy and safety of EUFLEXXA for treatment of painful osteoarthritis of the knee, with an open-label safety extension (the FLEXX trial). Semin Arthritis Rheum.

[73] Baraf HS, Strand V, Hosokawa H, Akahane O, Lim S, Yaguchi M (2009). Effectiveness and safety of a single intraarticular injection of GEL-200, a new cross-linked formulation of Hyaluronic Acid [HA] in the treatment of symptomatic osteoarthritis [OA] of the knee. Osteoarthr Cartil.

[74] Dickson DJ, Hosie G, English JR (2001). A double-blind, placebo-controlled comparison of hylan G-F 20 against diclofenac in knee osteoarthritis. J Clin Res.

[75] Puhl W, Bernau A, Greiling H, Kopcke W, Pforringer W, Steck KJ, Zacher J, Scharf HP (1993). Intra-articular sodium hyaluronate in osteoarthritis of the knee: a multicenter, double-blind study. Osteoarthritis Cartilage.

[76] Koog YH, Lee JS, Wi H (2014). Nonspecific Adverse Events in Knee Osteoarthritis Clinical Trials: A Systematic Review. PloS One.

[77] Smith SM, Wang AT, Katz NP, McDermott MP, Burke LB, Coplan P, Gilron I, Hertz SH, Lin AH, Rappaport BA (2013). Adverse event assessment, analysis, and reporting in recent published analgesic clinical trials: ACTTION systematic review and recommendations. Pain.

