# Supplementary material for: Hyaluronic acid injection therapy for osteoarthritis of the knee: concordant efficacy and conflicting serious adverse events in two systematic reviews
Source: Syst Rev. 2016 Nov 4;5:186. doi: 10.1186/s13643-016-0363-9 (PMC5097414; doi:10.1186/s13643-016-0363-9)
Supplement: Additional file 1: Table S1. — Search methodology. (DOC 29 kb) [file 13643_2016_363_MOESM1_ESM.doc]

**Appendix 1: Search Methodology**

========================================================================

**DATABASE SEARCHED & TIME PERIOD COVERED:**

PUBMED – 1/1/1990-12/11/2014

**SEARCH STRATEGY:**

(hyaluronic acid OR hyaluronate* OR hyaluronan OR hylan) AND (osteoarthritis, knee OR (knee* AND osteoarthritis) OR (knee* AND arthrit*) OR gonarthrosis)

OR

(hyaluronic acid OR hyaluronate* OR hyaluronan OR hylan) AND degenerative joint disease AND (knee OR knees)

OR

((viscosupplement* OR visco-supplement*) AND (osteoarthritis, knee OR (knee* AND osteoarthritis) OR (knee* AND arthrit*) OR gonarthrosis))

========================================================================

**DATABASE SEARCHED & TIME PERIOD COVERED:**

Web of Science Indexes=SCI-EXPANDED, SSCI, A&HCI, CPCI-S, CPCI-SSH – 1/1/1990-12/11/2014

**SEARCH STRATEGY:**

TS=(hyaluronic acid OR hyaluronate* OR hyaluronan OR hylan) AND TS=(knee OR knees) AND TS=(osteoarthritis OR arthrit* OR gonarthrosis)

OR

TS=(hyaluronic acid OR hyaluronate* OR hyaluronan OR hylan) AND TS=(knee OR knees) AND TS=(degenerative joint disease)

OR

TS=(viscosupplement* OR visco-supplement*) AND TS=(knee OR knees) AND TS=(osteoarthritis OR arthrit* OR gonarthrosis)

========================================================================

**DATABASE SEARCHED & TIME PERIOD COVERED:**

Scopus – 1/1/1990-12/12/2014

**SEARCH STRATEGY:**

TITLE-ABS-KEY ( hyaluronic acid OR hyaluronate* OR hyaluronan OR hylan ) AND TITLE-ABS-KEY ( knee OR knees )

OR

TITLE-ABS-KEY ( hyaluronic acid OR hyaluronate* OR hyaluronan OR hylan ) AND TITLE-ABS-KEY ( knee OR knees ) AND ALL ( "degenerative joint disease" )

OR

TITLE-ABS-KEY ( viscosupplement* OR visco-supplement* ) AND TITLE-ABS-KEY ( knee OR knees ) AND TITLE-ABS-KEY ( osteoarthritis OR arthrit* OR gonarthrosis )

========================================================================

**DATABASE SEARCHED & TIME PERIOD COVERED:**

Cochrane – 1/1/1990-12/12/2014

**SEARCH STRATEGY:**

**SEARCH 1:**

hyaluronic acid or hyaluronate* or hyaluronan or hylan:ti,ab,kw (Word variations have been searched)

AND

osteoarthritis, knee or (knee* and osteoarthritis) or (knee* and arthrit*) or gonarthrosis

**SEARCH 2:**

hyaluronic acid or hyaluronate* or hyaluronan or hylan:ti,ab,kw (Word variations have been searched)

AND

degenerative joint disease

AND

knee or knees

**SEARCH 3:**

viscosupplement* or visco-supplement*:ti,ab,kw (Word variations have been searched)

AND

osteoarthritis, knee or (knee* and osteoarthritis) or (knee* and arthrit*) or gonarthrosis

========================================================================

**DATABASE SEARCHED & TIME PERIOD COVERED:**

Embase– 1/1/1990-12/12/2014

**SEARCH STRATEGY:**

**SEARCH 1:**

hyaluronic NEAR/2 acid* OR hyaluronate* OR 'hyaluronan' OR 'hyaluronan'/exp OR hyaluronan OR hylan AND

'osteoarthritis' OR 'osteoarthritis'/exp OR osteoarthritis AND ('knee' OR 'knee'/exp OR knee OR knees) OR (knee* AND arthrit*) OR 'gonarthrosis' OR 'gonarthrosis'/exp OR gonarthrosis

AND

Humans/lim

**SEARCH 2:**

hyaluronic NEAR/2 acid* OR hyaluronate* OR 'hyaluronan' OR 'hyaluronan'/exp OR hyaluronan OR hylan

AND

degenerative AND ('joint' OR 'joint'/exp OR joint) AND ('disease' OR 'disease'/exp OR disease)

AND

'knee' OR 'knee'/exp OR knee OR knees

AND

Humans/lim

**SEARCH 3:**

viscosupplement* OR 'visco supplement' OR 'visco supplements' OR 'visco supplemental'

AND

'osteoarthritis' OR 'osteoarthritis'/exp OR osteoarthritis OR arthrit* OR 'gonarthrosis' OR 'gonarthrosis'/exp OR gonarthrosis

AND

'knee' OR 'knee'/exp OR knee OR knees

AND

Humans/lim

**==========================================================================**

**DATABASE SEARCHED & TIME PERIOD COVERED:**

New York Academy of Medicine Grey Literature Report – Earliest dates to 12/12/2014

**SEARCH STRATEGY:**

Hyaluronic OR hyaluronate OR hyaluronan OR hylan OR viscosupplement OR visco-supplement

**==========================================================================**

**DATABASE SEARCHED & TIME PERIOD COVERED:**

Canadian Agency for Drugs and Technologies in Health (CADTH) – Earliest-12/12/2014

**SEARCH STRATEGY:**

hyaluronic OR hyaluronate OR hyaluronan OR hylan OR viscosupplement OR visco-supplement

**==========================================================================**

Food and Drug Administration Premarket Approval database– Earliest-12/12/2014

**==========================================================================**

ClinicalTrials.gov– Earliest-12/12/2014
