# Supplementary material for: Hyaluronic acid injection therapy for osteoarthritis of the knee: concordant efficacy and conflicting serious adverse events in two systematic reviews
Source: Syst Rev. 2016 Nov 4;5:186. doi: 10.1186/s13643-016-0363-9 (PMC5097414; doi:10.1186/s13643-016-0363-9)
Supplement: Additional file 2: Table S2. — Evidence table. (DOCX 91 kb) [file 13643_2016_363_MOESM2_ESM.docx]

**Appendix 2: Evidence Table**

| **Author, Year** | **Study Location, Years, Name, Design, and Funding** | **Participants** | **Inclusion criteria** | **Exclusion criteria** | **Comorbidities** | **Study Arms** |
| --- | --- | --- | --- | --- | --- | --- |
| Altman et al., 1998[52] | US  <1998  RCT/CCT parallel  Funding: Industry | Age Range: 40-90  Mean age: 64  SD controls: 10 (whole group)  Number of participants enrolled: 495  Number of participants in analysis: 333  Number of knees: NR  Mean BMI: NR  % Female: 57 | Diagnosis of osteoarthritis of the knee: ACR(NR) Knee radiograph(Kellgren-Lawrence grade 2 or 3, >=1 osteophyte)  Duration of symptoms Knee pain 1 year or more  Score(s) on OA assesments  VAS for pain on a 50 foot walk: >=20mm  WOMAC pain subscale: >=20 on >=1 item out of 5  6-point categorical scale: moderate or marked main  Minimum age: 40  Maximum age: 80  No prior IA HA injection within one year  No other IA injections, including corticosteroids within preceding 3 months  If both knees affected, more serious one was used | NR | NR | Arm 1: N = 115 Mean age: 65 (10) Placebo/sham Acetaminophen up to 4000mg /day permitted as rescue  Arm 2: N = 105 Mean age: 62(10) Hyalgan 20mg/2ml Molecular weight: 500-730kD Oral placebo for naproxen twice daily and Acetaminophen up to 4000mg /day permitted as rescue  Arm 3: N = 113 Mean age: 63(9) NSAID  Total treatments: 5 Time between treatment: 1 week |
| Berenbaum et al., 2012[53] | France, Germany  2008-2009  EudraCt no 2008-003875-35  RCT/CCT parallel  Funding: Industry | Age Range: NR  Mean age: 67  SD controls: NR  Number of participants enrolled: 426  Number of participants in analysis: 426  Number of knees: NR  Mean BMI: 27.7 (3.1) for Hyalgan  % Female: 63 | Diagnosis of osteoarthritis of the knee: ACR(NR) Radiologic(Kellgren-Lawrence stage II or III within past 12 months)  Duration of symptoms At least 6 months  Failure of another treatment modality: Analgesics and/or regular NSAIDs  Score(s) on OA assesments  Global knee pain VAS: 40mm/100mm  WOMAC pain subscale score: 25 or greater on the 0-100 normalized scale  Lequesne Index: 4 or greater  Minimum age: 49  Maximum age: 81  Intolerance to NSAIDs or weak opioids  Radiologic evidence of bilateral OA if global pain VAS in contralateral knee<30mm | Current or prior receipt of HA in affected knee  Current or prior receipt of glucocorticoids in affected knee  Use of certain analgesics: Opioids within past month of baseline  Other musculoskeletal or joint disease or condition that limits mobility: Inflammatory or other rheumatic diseases  Patelofemoral symptomatic OA  Secondary OA  Symptomatic hip OA ipsilateral to target knee  Clinical joint effusion  Excessive varus or valgus knee deformity (on physical exam, confirmed radiographically |  | Arm 1: N = 209 Mean age: 66.1 (8.1) Hyalgan Molecular weight: 500 kD-730 kD NSAID or paracetamol up to 4g/d as rescue medication  Arm 2: N = 217 Mean age: 67.2 (7.8) GO-ON (2.5 ml, 10mg/ml) Molecular weight: 800 kD-1500 kD NSAID or paracetamol up to 4g/d as rescue medication  Total treatments: 3 Time between treatment: 3 weeks |
| Blanco et al., 2008[29] | Spain  <2008  RCT/CCT parallel  Funding: Industry | Mean age: 68.3  SD controls: (9.1)  Number of participants enrolled: 42  Mean BMI: 33  % Female: 76 | Diagnosis of osteoarthritis of the knee: WOMAC pain(>/=150mm) ACR Kellgren-Lawrence(IV)  Minimum age: 40  Waiting list for knee replacement | Prior surgical procedure on affected knee  Current or prior receipt of HA in affected knee  Current or prior receipt of glucocorticoids in affected knee  Other musculoskeletal or joint disease or condition that limits mobility  Use of glucosamine within prior 3 months  Use of an investigational drug within 30 days of study entry  CNS impairment, impaired coagulation  Known sensitivity to HA, paracemtamol, or diclofenac  Immune-comprised or receiving immuno-suppressive therapy or considered unable to complete treatment or followup | NR | Arm 1: N = 20 Mean age: 68.3(9.1) Placebo/sham Paracetamol and/or dicofenac as rescue analgesics  Arm 2: N = 22 Mean age: 67.5(8.1) Adant Molecular weight: 900 kDa  Total treatments: 10 (2 cycles of 5 weekly injections, separated by 24 weeks) Time between treatment: 1 week |
| Brandt et al., 2001[20] | US  1996-1997  RCT/CCT parallel  Funding: Industry | Age Range: NR  Mean age: NR  SD controls: NR  Number of participants enrolled: 226  Number of participants in analysis: 226 and 135  Number of knees: NR  Mean BMI: HA: 32(6); Saline: 30.1(6.2)  % Female: 63 | Diagnosis of osteoarthritis of the knee: Kellgren-Lawrence(Grade II or III)  Score(s) on OA assesments  WOMAC: pain score 13 or greater in knee to be treated knee and less than 13 in contralateral knee  Minimum age: 50  Willing to d/c other analgesics and NSAIDs for 5 half-lives of the relevant drug  Able to walk 50 feet unassisted  Not pregnant or planning pregnancy | Prior surgical procedure on affected knee: Arthroplasty  Current or prior receipt of HA in affected knee  Current or prior receipt of glucocorticoids in affected knee  Initiation of quadriceps exercise program within 4 months of screening  Kellgren-Lawrence Grade IV radiographic changes in either knee  Tx with anticoagulants, immunosuppressives, or muscle relaxants  Inability to tolerate acetaminophen  Clinically significant comorbidity (renal or hepatic disease) or abnormality in routine lab tests or allergy to lidocaine | Involvement of both knees: HA: 78% Saline: 88% | Arm 1: N = 112 Mean age: 67(8.4) Placebo/sham  Arm 2: N = 114 Mean age: 65(8.4) Orthovisc (2 mL, 15mg/mL) Molecular weight: 1000-2900 kD (considered high MW)  Total treatments: 3 Time between treatment: 1 week |
| DeCaria et al., 2012[21] | Ontario Canada  <2012  RCT/CCT parallel  Funding: Non-industry | Age Range: 60-80  Mean age: NR  SD controls: NR  Number of participants enrolled: 30  Number of participants in analysis: na  Number of knees: na  Mean BMI: HA: 30.48(6.16) Placebo: 29.40(4.11)  % Female: 47 | Diagnosis of osteoarthritis of the knee: ACR: Kellgren-Lawrence(Grade II-III) ACR clinical criteria for knee pain(NR)  Failure of another treatment modality: Multiple years  Minimum age: 60  Maximum age: 80 | Prior surgical procedure on affected knee: Except for arthroscopy 18 months or more before study commencement  Other musculoskeletal or joint disease or condition that limits mobility: Non OA arthritis (e.g., RA, gout), OA in other lower limbs, end-stage knee OA; lower back pathology that limited walking, leg length differential>2cm  A neurological or cardiovascular condition that could impair gait function  Cognitive impairment  Intraarticular injection within 6 months prior to study commencement  Chronic use of oral corticosteroids | NR | Arm 1: N = 15 Mean age: 72.93 (5.48) 500 mg acetaminophen to be taken up to 4g/day as rescue medication Placebo/sham 1.2 ml 0.001 mg/ml inert HA  Arm 2: Hyaluronic acid (2 ml, 20 mg/ml) Molecular weight: 730 kD 500 mg acetaminophen to be taken up to 4g/day as rescue medication  Total treatments: 3 Time between treatment: 1 week |
| Dixon et al., 1988[50] | UK  <1988  RCT/CCT parallel  Funding: Industry | Age Range: 43-85  Mean age: 68.5  SD controls: NR  Number of participants enrolled: 63  Number of participants in analysis: 53  Number of knees: NR  Mean BMI: NR  % Female: 54 | Diagnosis of osteoarthritis of the knee: Symptomatic OA NR | Use of certain analgesics: If other than for OA  Other musculoskeletal or joint disease or condition that limits mobility: Hip OA; primary inflammation of the knee (e.g., RA, psoriatic arthropathy, pseudogout, joint infection)  Skin conditions overlying the joint  Poor general health | NR | Arm 1: N = 33 Mean age: nr Hyalgan 0.2mg/2 ml Molecular weight: NR Placebo/sham  Arm 2: N = 30 Mean age: nr Hyalgan 20mg/2 ml Molecular weight: NR Paracetamol was permitted but NSAIDS, corticosteroids, and strong analgesics were not  Total treatments: Varied 1 for first 3 weeks and then 2 |
| Dougados et al., 1993[22] | France  <1993  RCT/CCT parallel  Funding: NR | Mean age: 69.0  SD controls: 10.6  Number of participants enrolled: 110  Number of participants in analysis: 95 (ITT also done)  Mean BMI: NR  % Female: 71.0 | Diagnosis of osteoarthritis of the knee: ACR  Score(s) on OA assesments  VAS for pain: >=40 out of 100  Femorotibial localization  Knee effusion | Prior surgical procedure on affected knee: Prosthesis or any intra-articular surgery during the preceding 2 years  Use of certain analgesics: Dose of NSAIDS or analgesics stable during previous month  Other musculoskeletal or joint disease or condition that limits mobility: Secondary osteoarthritis of the knee  Serious concomitant medical illness  Any arthrocentesis during prior 3 months  Stable dose of any basic OA therapy stable for prior 3 months;  Stable use of any physiotherapy during the previous month and first 7 weeks of study | NR | Arm 1: Placebo/sham  Arm 2: Hyalectin (Hyalgan) Molecular weight: 500-730 kDa  Total treatments: 4 Time between treatment: 1 week |
| Forster et al., 2003[32] | UK  <2002  RCT/CCT parallel  Funding: NR | Age Range: NR  Mean age: 61.5  SD controls: NR  Number of participants enrolled: 38  Number of participants in analysis: 32  Mean BMI: NR  % Female: NR | Diagnosis of osteoarthritis of the knee:  On waiting list for arthroscopic washout  Fitness for general or local anesthesia | Prior surgical procedure on affected knee  Current or prior receipt of HA in affected knee  Current or prior receipt of glucocorticoids in affected knee  Mechanical symptoms | NR | Arm 1: N = 19 Mean age: 63 Arthroscopic washout  Arm 2: N = 19 Mean age: 60 Hyalgan Molecular weight: 500-730 kD  Total treatments: 5 Time between treatment: 1 week |
| Grecomoro et al., 1987[51] | Italy  <1987  RCT/CCT parallel  Funding: NR | Age Range: 43-92  Mean age: 64.88  SD controls: 10.94  Number of participants enrolled: 34  Number of knees: 40  % Female: 19/34 | Diagnosis of osteoarthritis of the knee: Not reported  Knee pain with movement | NR | Involvement of both knees: 6/34 | Arm 1: N = 20 knees Mean age: NR Placebo/sham  Arm 2: N = 20 knees Mean age: NR Hyalgan Molecular weight: 500K-750K  Total treatments: 3 Time between treatment: 1 week |
| Henderson et al., 1994[54] | UK  <1994  RCT/CCT parallel  Funding: NR | Age Range: NR  Mean age: NR  SD controls: NR  Number of participants enrolled: 91  Number of participants in analysis: 84  Number of knees: NR  Mean BMI: NR  % Female: 69 | Diagnosis of osteoarthritis of the knee: Clinical history and radiological evidence Kellgren-Lawrence(Grades I and II: severity group 1; Grades III and IV: severity group 2)  Score(s) on OA assesments  VAS scale for pain evoked by activities: minimum score of 30 mm out of 100mm | Other musculoskeletal or joint disease or condition that limits mobility: Inflammatory joint disease, metabolic bone disease, anserine bursitis, pain referred from other structures | Involvement of both knees: >99% | Arm 1: N = 20 (Severity group I) Mean age: 60.0(1.9) Placebo/sham  Arm 2: N = 26 Severity Group 2 Second placebo group  Arm 3: N = 18 Severity Group 1 Mean age: 63.9(1.9) Hyalgan (20mg/2mL) Molecular weight: NR  Arm 4: N = 26 Severity Group 2 Mean age: 67.0(1.7) Hyalgan  Total treatments: 5 Time between treatment: 1 week |
| Huang et al., 2011[23] | Taiwan  <2011  RCT/CCT parallel  Funding: Industry | Age Range: nr  Mean age: 65.0  SD controls: 8.3  Number of participants enrolled: 200  Number of participants in analysis: 198  Number of knees: NR  Mean BMI: 25.6(3.6)  % Female: 76 | Diagnosis of osteoarthritis of the knee: ACR (knee pain with one or more of the following conditions: age>50, crepitus, or morning stiffness<30 minutes duration Radiographic evidence(Kellgren Lawrence score II and III, predominance in tibio-femoral compartment) VAS pain scores on 50-foot walking test(>=40mm)  Minimum age: 50  Any acute disease or trauma leading to secondary OA had to have occurred at least 5 years before study entry | Current or prior receipt of glucocorticoids in affected knee  Other musculoskeletal or joint disease or condition that limits mobility  Severe degeneration of knee joint with marked joint narrowing, varus, or valgus deformity of the knee (>12") or other joint deformities or other joint disorders  Joint or skin infections  Joint prostheses of lower limb or symptomatic hip  Inflammatory joint disease, specific arthropathy, severe axis deviations or instabilities, | NR | Arm 1: N = 100 Mean age: 64.2(8.4) Placebo/sham  Arm 2: N = 100 Mean age: 65.9(8.1) Hyalgan (20mg/2ml)  Total treatments: 5 Time between treatment: 1 week |
| Huskisson et al., 1999[24] | United Kingdom  <1997  RCT/CCT parallel  Funding: NR | Age Range: nr  Mean age: nr  SD controls: nr  Number of participants enrolled: 100  Number of participants in analysis: 81  Number of knees: NR  Mean BMI: nr  % Female: 67 | Diagnosis of osteoarthritis of the knee: ARA Criteria Kellgren-Lawrence(II or III)  Consistent pain for 3 months  Moderate to severe pain on walking | Current or prior receipt of glucocorticoids in affected knee  Other musculoskeletal or joint disease or condition that limits mobility: Serious functional impairment at the knee, hip OA, other related joint OA, psoriasis, sacroiliitis, painful knee conditions other than OA  Kellgren Lawrence IV  Known or suspected joint infection  Poor general health or other conditions which would prevent regular hospital attendance  Skin conditions overlying the joint  Severe intercurrent hepatic or renal disease or major general medical conditions |  | Arm 1: N = 50 Mean age: 64.8 (9.3) Placebo/sham  Arm 2: N = 50 Mean age: 65.8 (8.8) Hyalgan Molecular weight: 500-730 kDa  Total treatments: 5 Time between treatment: 1 week |
| Kahan et al., 2003[55] | France  10/1998-2/2000  RCT/CCT parallel  Funding: NR | Mean age: 66  SD controls: 10  Number of participants enrolled: 506  Number of participants in analysis: 506  Mean BMI: 28  % Female: 67.5 | Diagnosis of osteoarthritis of the knee: ACR Kellgren-Lawrence(any)  Failure of another treatment modality: Two courses of NSAID therapy, each at least 10 d long, within the last 3 months and/or a symptomatic slowacting drug taken continuously during the last 2 months  Score(s) on OA assesments  Pain VAS: >= 40 mm / 100 mm  Minimum age: 18 | Prior surgical procedure on affected knee: Arthroscopy, lavage, meniscectomy, etc.) within the last year; TKR ever  Current or prior receipt of HA in affected knee  Current or prior receipt of glucocorticoids in affected knee  Inflammatory flare in the target knee (effusion with nocturnal pain, local heat or redness, morning stiffness for longer than 45 min or greater than 50% increase in the VAS pain score as compared to the previous week)  Synovectomy, tibial osteotomy  Surgery scheduled within last 9 months | Involvement of both knees: 74 | Arm 1: N = 253 Mean age: 66 (10) Conventional treatment  Arm 2: N = 253 Mean age: 66 (10) Synvisc Molecular weight: NR  Total treatments: 3 Time between treatment: 1 week |
| Karlsson et al., 2002[25] | Sweden  <2002  RCT/CCT parallel  Funding: Industry | Age Range: NR  Mean age: reported by arm below  SD controls: reported by arm below  Number of participants enrolled: 246  Number of participants in analysis: ITT: 246 PP: 210  Number of knees: NR  Mean BMI: 28  % Female: 61 | Score(s) on OA assesments  Lequesne algofunctional index: >=10  Weight-bearing pain VAS: >=40mm  Minimum age: 60  Normal general physical exam | Prior surgical procedure on affected knee: Arthroscopy, arthrography, surgery less than 6 months prior to inclusion  Current or prior receipt of HA in affected knee  Current or prior receipt of glucocorticoids in affected knee  Other musculoskeletal or joint disease or condition that limits mobility: RA or other inflammatory joint disease (ACR criteria)  Any disabling problem of the musculoskeletal system or other organ system which could interfere with the assessment of efï¬cacy  Bone attrition in either knee (Ahlback III or IV), previous intra-articular fracture of the knee  Alcohol or drug abuse  Known allergy to any substance related to the study  Clinically relevant hematological of known clinical chemistry values outside the reference values at the time of inclusion  Any disabling problem with any other organ system that could interfere with the assessment of efficacy | NR | Arm 1: N = 66 (57 PP) Mean age: 71(6) Placebo/sham  Arm 2: N = 92 (76 PP) Mean age: 72(7) Artzal (2.5 ml 1% hyaluronan) Molecular weight: 1,000 kDa  Arm 3: N = 88 (77 PP) Mean age: 70(7) Synvisc (2 ml 0.8%) Molecular weight: 7,000 kDa  Total treatments: 3 Time between treatment: 1 day |
| Khanasuk et al., 2012[30] | Thailand  2011-2012  RCT/CCT parallel  Funding: NR | Mean age: NR  SD controls: NR  Number of participants enrolled: 32  Number of participants in analysis: 30  Number of knees: NR  Mean BMI: 26  % Female: 80 | Diagnosis of osteoarthritis of the knee: ACR for primary OA of the knee(NR) Pain VAS(>=3/10) Kellgren-Lawrence radiological grading(>=Grade II)  Minimum age: 45 | Current or prior receipt of HA in affected knee  Intention to take pain medication after injection  History of allergy to avian products | NR | Arm 1: N = 15 Mean age: 65.1(9.6) Hylan GF-20 (Synvisc)(single 6 ml injection) Molecular weight: Reported as High  Arm 2: N = 15 Mean age: 67.0(9.5) Hyalgan (single injection Molecular weight: Reported as Low  Total treatments: 1 |
| Leopold et al., 2003[56] | US  2000-2002  RCT/CCT parallel  Funding: Non-industry | Age Range: 39-83  Mean age: NR  SD controls: NR  Number of participants enrolled: 100  Number of participants in analysis: 80  Number of knees: NR  Mean BMI: CS: 29.3 HA:28.8  % Female: CS: 56 HA: 52 | Diagnosis of osteoarthritis of the knee: Radiographic evidence of symptomatic knee OA(NR)  Minimum age: 18  Dissatisfaction with prior attempts at nonoperative management modalities | Current or prior receipt of HA in affected knee  Other musculoskeletal or joint disease or condition that limits mobility  Pregnant or lactating  Radiographic evidence of bone on bone arthritis or Chodrocalcinosis  Insufficiency of the collateral ligament, of the ACL or PCL with concomitant symptomatic giving way of the affected extremity or a current infection in the affected extremity as demonstrated on physical exam  History of crystalline arthropathy or neuropathic arthropathy  Allergy or hypersensitivity to any of the study medications or to poultry products | NR | Arm 1: N = 42 Mean age: 64  Arm 2: N = 38 Mean age: 66 Hylan G-F 20 (16mg/2ml)  Total treatments: 3 HA 1CS Time between treatment: 1 week |
| Lundsgaard et al., 2008[26] | Denmark  <2008  NCT00144820  RCT/CCT parallel  Funding: Non-industry | Age Range: NR  Mean age: 69.6  SD controls: 7.27  Number of participants enrolled: 251  Number of participants in analysis: 243  Mean BMI: 29.3  % Female: 52.4 | Score(s) on OA assesments  Daily knee pain on VAS (that did not respond to analgesics): 20mm/100mm  Minimum age: 59  Daily | Prior surgical procedure on affected knee: Invasive procedures within past 6 months  Current or prior receipt of glucocorticoids in affected knee  Other musculoskeletal or joint disease or condition that limits mobility: RA or other inflammatory arthritis  Contra-indication to hyaluronate  Contra-indication to knee injection  Medications that could interfere with intervention  Comorbidity, e.g. psychosis or dementia that could interfere  Knee infection or uric acid crystals | NR | Arm 1: Saline 2ml  Arm 2: Saline 20 mL, no hyaluronate  Arm 3: Hyalgan Molecular weight: NR  Total treatments: 4 Time between treatment: 1 week |
| Pavelka et al., 2011[57] | Czech Republic, France, Italy, Switzerland, the Slovak Republic and Germany  November 2007 - January 2009  NCT00556608  RCT/CCT parallel  Funding: Industry | Age Range: 41-80  Mean age: 65  SD controls: 9  Number of participants enrolled: 381  Number of participants in analysis: 354  Mean BMI: 27  % Female: 72.9 | Diagnosis of osteoarthritis of the knee: ACR Kellgren-Lawrence(2 or 3)  Duration of symptoms 3 months  Failure of another treatment modality: NSAIDS  Score(s) on OA assesments  WOMAC pain: include 40 - 80  Minimum age: 40 | Prior surgical procedure on affected knee: TKR, arthroplasty  Current or prior receipt of HA in affected knee  Current or prior receipt of glucocorticoids in affected knee  Use of certain analgesics: Chronic use of NSAIDS, analgesics or narcotics  Other musculoskeletal or joint disease or condition that limits mobility: Pain mainly related to femoral patellar syndrome at the target knee, no remaining joint space width at the target knee, symptomatic hip osteoarthritis or other condition that would interfere with study assessments, severe varus/valgus deformity in the target knee, history or current evidence of other joint diseases, such as inflammatory, infective or metabolic joint disease, concomitant rheumatic disease, significant injur  Lymphatic stasis in the relevant limb, skin infection, disease or trauma at the injection site  Initiation of target knee physical therapy in the past 3 months, initiation/ change in dose of symptomatic slow-acting drugs for osteoarthritis  BMI >=32 | Involvement of both knees: 66% | Arm 1: N = 192 Mean age: 65.1 (9.1) Synovial Molecular weight: 800 - 1,200 kD  Arm 2: N = 188 Mean age: 64.9 Synvisc Molecular weight: 6,000 kD  Total treatments: 3 Time between treatment: 1 week |
| Petrella et al., 2002[27] | Canada  < 2002  RCT/CCT parallel  Funding: Industry | Age Range: NR  Mean age: 65.5  SD controls: 9.5  Number of participants enrolled: 120  Number of participants in analysis: 108  Mean BMI: 30.7  % Female: 45.8 | Diagnosis of osteoarthritis of the knee: Kellgren-Lawrence(1 to 3 included)  Score(s) on OA assesments  VAS pain 0-10 scale: 3+  Unilateral OA | Current or prior receipt of HA in affected knee  Current or prior receipt of glucocorticoids in affected knee  NSAID intolerance  Bilateral symmetric inflammatory reaction | Involvement of both knees: 0% | Arm 1: N = 28 Mean age: 62.6 (9.5) Placebo/sham  Arm 2: N = 25 Mean age: 67.3 (8.9) Suplasyn Molecular weight: NR Placebo pill  Arm 3: N = 29 Mean age: 65.0 (9.7) Suplasyn Molecular weight: NR NSAID  Arm 4: N = 26 Mean age: 66.3 (8.8) NSAID  Total treatments: 3 Time between treatment: 1 week |
| Petrella et al., 2008[58] | Canada  <2008  RCT/CCT parallel  Funding: Non-industry | Age Range: nr  Mean age: 71  SD controls: 8  Number of participants enrolled: 200  Number of participants in analysis: nr  Mean BMI: 27.2  % Female: 30 | Diagnosis of osteoarthritis of the knee: Medial compartment OA Radiographic grade(1-3)  Did not exhibit non-arthritis-related disease | Current or prior receipt of HA in affected knee  Current or prior receipt of glucocorticoids in affected knee  End-stage OA in affected knee | NR | Arm 1: N = 50 Mean age: 71+/-8 Placebo/sham  Arm 2: N = 50 Mean age: 68+/-6 HA dual molecular weight Molecular weight: 580–780 kDa+1.2 to 2.0 million kDa  Arm 3: N = 50 Mean age: 69+/-5 HA low molecular weight Molecular weight: 500–730 kDa  Arm 4: N = 50 Mean age: 71+/9 HA high molecular weight Molecular weight: 6 million kDa  Total treatments: 3 Time between treatment: 1 week |
| Petrella et al., 2011[61] | Canada  < 2011  ISRCTN98630331  RCT/CCT parallel  Funding: NR | Age Range: NR  Mean age: 70  SD controls: 8  Number of participants enrolled: 200  Number of participants in analysis: Unclear  Mean BMI: 27  % Female: 57 | Diagnosis of osteoarthritis of the knee: Kellgren-Lawrence(1 to 3 included)  Score(s) on OA assesments  VAS pain: 45+ | Current or prior receipt of HA in affected knee  Current or prior receipt of glucocorticoids in affected knee  Use of certain analgesics: Dosage of glucosamine and/or chondroitin sulfate, and/or NSAIDs that has been stable over the preceding three months with the dosage remaining constant during the study  Other musculoskeletal or joint disease or condition that limits mobility: End stage OA  Active skin disease or infection in the area of the injection site  Any condition/ disease which in the opinion of the investigator could interfere with patient compliance and/ or interfere with the interpretation of the treatment results  Contra-indication to intra-articular injection or known hypersensitivity to Sodium Hyaluronate  Planned surgery on knee | NR | Arm 1: N = 50 Mean age: 71 (8) Placebo/sham  Arm 2: N = 50 Mean age: 68 (6) sodium hyaluronate Molecular weight: Combined high & low weight  Arm 3: N = 50 Mean age: 69 (5) sodium hyaluronate - low weight Molecular weight: 500-730 KDa  Arm 4: N = 50 Mean age: 71 (9) sodium hyaluronate - high weight Molecular weight: 6000 KDa  Total treatments: 3 Time between treatment: 1 week |
| Pham et al., 2004[28] | France  <2004  RCT/CCT parallel  Funding: NR | Age Range: 50+  Mean age: 64.9  SD controls: 7.7  Number of participants enrolled: 301  % Female: 65 average | Diagnosis of osteoarthritis of the knee: Presence of a symptomatic primary painful medial femorotibial knee OA defined by a daily pain visual analogue scale (VAS) score .30 mm in the previous month VAS for pain(>30mm in prior month) Joint space(>2mm) | Current or prior receipt of HA in affected knee  Current or prior receipt of glucocorticoids in affected knee  Use of certain analgesics: Diacerein or other antiinflammatories  Other musculoskeletal or joint disease or condition that limits mobility: Severe OA (jpint space <2mm), secondary knee OA, Paget's  Contraindications to HA  Need for surgery | NR | Arm 1: N = 85 Mean age: 64.9 (7.7) Placebo/sham  Arm 2: N = 131 Mean age: 71.0 NRD101 Molecular weight: 1.900 kDa  Arm 3: N = 85 Mean age: 64.5 Diacerein  Total treatments: 12? (3 course every 3 months for a year) Time between treatment: 1 week |
| Raman et al., 2008[31] | UK  < 2008  RCT/CCT parallel  Funding: Non-industry | Age Range: 42-82  Mean age: 67.2  SD controls: NR  Number of participants enrolled: 392  Number of participants in analysis: 380  Mean BMI: NR  % Female: 68 | Diagnosis of osteoarthritis of the knee:  Score(s) on OA assesments  VAS (10 point scale): 6+  Preferred tx strategy was viscosupplementation | Prior surgical procedure on affected knee  Current or prior receipt of HA in affected knee  Current or prior receipt of glucocorticoids in affected knee  Bilateral OA | NR | Arm 1: N = 199 Mean age: NR Synvisc (Hylan GF 20) Molecular weight: 6000 kD  Arm 2: N = 193 Mean age: NR Hyalgan Molecular weight: 500 - 730 kD  Total treatments: 3 for Synvisc, 5 for Hyalgan Time between treatment: 1 week |
| Roman et al., 2000[59] | Spain  < 2000  RCT/CCT parallel  Funding: NR | Age Range: 41-86  Mean age: 65.14  SD controls: 9.77  Number of participants enrolled: 49  Mean BMI: NR  % Female: 83.7 | Diagnosis of osteoarthritis of the knee: Kellgren-Lawrence(2 or 3 included) | NR | NR | Arm 1: N = 30 Mean age: NR Adant Molecular weight: 900 kD  Arm 2: N = 19 Mean age: NR Hyalgan Molecular weight: 800 kD  Total treatments: 5 Time between treatment: 1 week |
| Tamir et al., 2001[60] | Israel  < 2001  RCT/CCT parallel  Funding: NR | Age Range: NR  Mean age: 71  SD controls: NR  Number of participants enrolled: 49  Number of participants in analysis: Unclear  Mean BMI: NR  % Female: 73.5 | Diagnosis of osteoarthritis of the knee: Altman Kellgren-Lawrence(2 to 4 included)  Minimum age: 60  Maximum age: 85 | Prior surgical procedure on affected knee: No surgery ever, no arthroscopy within 6 months  Current or prior receipt of HA in affected knee  Current or prior receipt of glucocorticoids in affected knee  Other musculoskeletal or joint disease or condition that limits mobility: RA, other inflammatory arthritis, OA of hip, OA from fracture of knee  Skin conditions on knee | NR | Arm 1: N = 24 Mean age: 70 Placebo/sham  Arm 2: N = 25 Mean age: 71 Bio-Hy Molecular weight: 3000 kDa  Total treatments: 5 Time between treatment: 1 week |
