# Supplementary material for: Hyaluronic acid injection therapy for osteoarthritis of the knee: concordant efficacy and conflicting serious adverse events in two systematic reviews
Source: Syst Rev. 2016 Nov 4;5:186. doi: 10.1186/s13643-016-0363-9 (PMC5097414; doi:10.1186/s13643-016-0363-9)
Supplement: Additional file 3: Table S3. — Risk of bias assessment. (DOCX 958 kb) [file 13643_2016_363_MOESM3_ESM.docx]

**Appendix 3. Risk of Bias Assessment**

| ***Author, Year*** | ***Random sequence generation (selection bias)*** | ***Allocation concealment (selection bias)*** | ***Blinding of participants*** | ***Blinding of personnel/providers*** | ***Blinding of outcome assessors*** | ***Incomplete outcome data addressed (less than 20%)*** | ***Incomplete outcome data addressed (attrition bias) (Loss to follow-up missing or explained)*** | ***Selective reporting (reporting bias)*** | ***Other sources of bias (standardized measurement tool used)*** | ***Other sources of bias (washout period of at least 3 months for steroid injections or 6 months for Hyaluronic acid)*** | ***Other sources of bias (co-interventions either avoided in the trial design or did the authors ensure that they were similar between the index and control groups)*** | ***Complete outcome data*** |
| --- | --- | --- | --- | --- | --- | --- | --- | --- | --- | --- | --- | --- |
| Altman et al., 1998 |  |  |  |  |  |  |  |  |  |  |  |  |
| Berenbaum et al., 2012 |  |  |  |  |  |  |  |  |  |  |  |  |
| Blanco et al., 2008 |  |  |  |  |  |  |  |  |  |  |  |  |
| Brandt et al., 2001 |  |  |  |  |  |  |  |  |  |  |  |  |
| DeCaria et al., 2012 |  |  |  |  |  |  |  |  |  |  |  |  |
| Dixon et al., 1988 |  |  |  |  |  |  |  |  |  |  |  |  |
| Dougados et al., 1993 |  |  |  |  |  |  |  |  |  |  |  |  |
| Forster et al., 2003 |  |  |  |  |  |  |  |  |  |  |  |  |
| Henderson et al., 1994 |  |  |  |  |  |  |  |  |  |  |  |  |
| Huang et al., 2011 |  |  |  |  |  |  |  |  |  |  |  |  |
| Huskisson et al., 1999 |  |  |  |  |  |  |  |  |  |  |  |  |
| Kahan et al., 2003 |  |  |  |  |  |  |  |  |  |  |  |  |
| Karlsson et al., 2002 |  |  |  |  |  |  |  |  |  |  |  |  |
| Khanasuk et al., 2012 |  |  |  |  |  |  |  |  |  |  |  |  |
| Leopold et al., 2003 |  |  |  |  |  |  |  |  |  |  |  |  |
| Lundsgaard et al., 2008 |  |  |  |  |  |  |  |  |  |  |  |  |
| Pavelka et al., 2011 |  |  |  |  |  |  |  |  |  |  |  |  |
| Pham et al., 2004 |  |  |  |  |  |  |  |  |  |  |  |  |
| Petrella et al., 2002 |  |  |  |  |  |  |  |  |  |  |  |  |
| Petrella et al., 2011 |  |  |  |  |  |  |  |  |  |  |  |  |
| Raman et al., 2008 |  |  |  |  |  |  |  |  |  |  |  |  |
| Roman et al., 2000 |  |  |  |  |  |  |  |  |  |  |  |  |
| Tamir et al., 2001 |  |  |  |  |  |  |  |  |  |  |  |  |

Legend:

_Unclear=_

_Low risk of bias=_

_High risk of bias=_
