# Supplementary material for: Hyaluronic acid injection therapy for osteoarthritis of the knee: concordant efficacy and conflicting serious adverse events in two systematic reviews
Source: Syst Rev. 2016 Nov 4;5:186. doi: 10.1186/s13643-016-0363-9 (PMC5097414; doi:10.1186/s13643-016-0363-9)
Supplement: Additional file 4: Table S4. — Comparison of articles contributing to AE and SAE analyses in our review and the review by Rutjes and colleagues [8]. (DOCX 33 kb) [file 13643_2016_363_MOESM4_ESM.docx]

**Appendix 4: Comparison of articles contributing to AE and SAE analyses in our review and the review by Rutjes and colleagues.**[8]

| **Study** | **AEs included in our study** | **SAEs included in our study** | **SAEs included in Rutjes et al.** |
| --- | --- | --- | --- |
| Altman, 1998 | **X** | **X** |  |
| Altman, 2004 |  |  | **X** |
| Altman, 2009 |  |  | **X** |
| Anika, 2000 |  |  | **X** |
| Anika, 2001 |  |  | **X** |
| Baraf, 2009 |  |  | **X** |
| Blanco, 2008 |  |  | **X** |
| Berenbrau, 2012 | **X** |  |  |
| Brandt, 2001 | **X** | **X** |  |
| Dickson, 2001 |  |  | **X** |
| Dixon, 1988 | **X** | **X** |  |
| Dougados, 1993 | **X** | **X** |  |
| Genzyme, 2005 |  |  | **X** |
| Henderson, 1994 | **X** |  |  |
| Huang, 2011 | **X** | **X** | **X** |
| Huskisson, 1999 | **X** | **X** | **X** |
| Jubb, 2003 |  |  | **X** |
| Kahan, 2003 | **X** |  |  |
| Leopold, 2003 | **X** | **X** |  |
| Lundsgaard , 2008 | **X** | **X** |  |
| Neustadt, 2005 |  |  | **X** |
| Pavelka, 2011 | **X** |  |  |
| Pham, 2004 | **X** | **X** |  |
| Raynauld, 2002 |  |  | **X** |
| Raman, 2008 | **X** | **X** |  |
| Roman, 2000 | **X** |  |  |
| Sanofi-Aventis, 2010 |  |  | **X** |
| Tamir, 2001 | **X** |  |  |

Only two articles are included in both SAE analyses.
