# Supplementary material for: Hyaluronic acid injection therapy for osteoarthritis of the knee: concordant efficacy and conflicting serious adverse events in two systematic reviews
Source: Syst Rev. 2016 Nov 4;5:186. doi: 10.1186/s13643-016-0363-9 (PMC5097414; doi:10.1186/s13643-016-0363-9)
Supplement: Additional file 5: Table S5. — Definitions and counts of SAEs in the review by Rutjes and colleagues [8]. (DOCX 45 kb) [file 13643_2016_363_MOESM5_ESM.docx]

**Appendix 5: Definitions and counts of SAEs in the review by Rutjes and colleagues.[8]**

| **Study Author, Year** | **Number of SAEs included in analysis** | | **How SAEs were defined** | | **Notes** |
| --- | --- | --- | --- | --- | --- |
|  | **Treatment** | **Control** | **Treatment** | **Control** |  |
| Huskisson, 1999 | 2/50 | 1/50 | 1 cutaneous vasculitis, 1 skin reaction characterized by peeling of skin on hands and toes | Myocardial infarction | For all AE’s: “…reported adverse events which were not associated with local reactions in the knee joint area.”  Cutaneous vasculitis: “one in the HA group was felt to be severe and possibly treatment related. The patient developed a cutaneous vasculitis…. The event occurred during the first week of treatment and progressed during the course of the study. The patient did not, however, interrupt the study.”  Skin reaction: “One other adverse event, which occurred in the HA group 8 days after the last injection, was considered severe and possibly drug related. This was a skin reaction…”  MI: “In the placebo group, there were no adverse events considered to be related to treatment.” |
| Anika, 2000 | - | - |  |  | Not available to us |
| Anika, 2001 | - | - |  |  | Not available to us |
| Dickson, 2001 | 2/50 | 1/54 |  |  | Unable to locate article, authors not responsive |
| Raynauld, 2002 | 0/127 | 1/128 | N/A | Gastro-duodenal ulcer | “There was one serious adverse event in the AC group (patient presented to the emergency room with a gastro-duodenal ulcer) listed by the investigator as remotely related to appropriate care.” |
| Jubb, 2003 | 27/208 | 14/200 | “SAE” 1 death from myocardial infarction | “SAE” | “All serious SAEs were considered by the investigators to be the result of concomitant disease and not to be drug related” |
| Altman, 2004 | 7/173 | 3/174 | “SAE” | “SAE” | “SAEs were judged treatment-unrelated serious adverse events” “Treatment-related SAEs were zero in each group” |
| Genzyme, 2005 | - | - |  |  | Not available to us |
| Neustadt, 2005 | 4/247 | 3/123 | “Potentially serious AE” | “Potentially serious AE” | “Potentially serious AEs included angina, myocardial infarction, gastrointestinal hemorrhage, and gastrointestinal cancer. None of the serious adverse events were considered by the investigators to be related to treatment” |
| Blanco, 2008 | 1/25 | 1/23 | “SAE” | “SAE” | From methods: “An assessment of the relationship of adverse events to study medication was also conducted. Naranjo's algorithm was used to determine the degree of causality.” Naranjo, et al. A method for estimating the probability of adverse drug reactions. Clin Pharmacol Ther.  From results: “Related adverse events (AE) did not occur in either the placebo group or the HA group.” |
| Altman, 2009 | 9/293 | 9/295 | “Treatment Emergent Adverse Event” | “Treatment Emergent Adverse Event” | The most common serious treatment emergent adverse events were pneumonia and TIA (each affecting 2 subjects). None of the serious treatment emergent adverse events were considered related to study treatment” |
| Baraf, 2009 | 8/249 | 0/128 | 4 “Serious adverse events”, 4 cancer | N/A | “Serious AEs occurred in 8 subjects; including 4 cancers: all receiving Gel-200, none related to study injection without evidence to support a causal relationship.” |
| Sanofi-Aventis, 2010 | 2/79 | 6/80 | “SAE” | “SAE” | Eight subjects experienced SAEs: 2 subjects in the HYALGAN group and 6 subjects in the PB-saline group; none of the SAEs were considered device related. |
| Huang, 2011 | 3/100 | 2/98 | 1 forearm fracture, 1 intestinal obstruction, 1 aggravated urinary incontinence | 1 upper gastrointestinal bleeding, 1 joint sprain | “All were considered to be unrelated to study treatment.” |

Few articles broke out counts by group or provided details on the kinds of events that were considered serious.

**Appendix 6: Comparison of AE and SAE categorization in our review and the review by Rutjes and colleagues.[8]** Both articles categorized AEs as serious or non-serious. Our review also categorized by locality (joint, non-joint local, or other). Both reviews omitted some SAEs that were qualitatively similar to SAEs counted by the other group. Our review excluded events only that did not describe the type of SAE that occurred. Rutjes et al. omitted some events that were similar to events they did count as SAEs.

| **AEs included in AE and SAE analyses in our review** | **AEs included in AE and SAE analyses by Rutjes et al.** |
| --- | --- |
| **Serious, joint**  acute synovitis  flare at the knee joint  haemarthrosis  post-injection flares  severe knee swelling and/or effusion  **Serious, local**  [none]  **Serious, other**  influenza-like symptoms  skin disorders  death  fever  forearm fracture  intestinal obstruction  myocardial infarction  skin reaction (peeling on hands and toes, erythema)  upper gastrointestinal bleeding  **Not serious, joint**  knee pain during or after injection  arthralgia  effusion  increase in effusion volume  knee pain after injection related to the injection procedure  local joint pain and swelling  musculoskeletal  stiffness in the index knee  transient increase in pain/swelling in the treated knee  **Not serious, local**  cutaneous vasculitis  erythema at the injection site  injection site pain  injection site reactions  local reaction at the injection site with pain, tenderness and erythema  local reactions at the injection site  local skin (ecchymosis and rash)  minor discomfort during the injection  pain and local swelling at the injection site  pain and swelling  painful injection  pruritis (local)  skin  **Not serious, other**  central and peripheral nervous disorders (headache, vertigo)  diarrhea  gastralgia  nausea, vomiting  other gastrointestinal disorders  respiratory system disorders (bronchitis, rhinitis…)  urine coloration  aggravated urinary incontinence  gastrointestinal  gastrointestinal complaints  general body  headache  joint sprain  phlebitis  pruritus  **Omitted from our analysis (due to lack of specificity in AE or SAE description in original article)**  “adverse events not associated with local reactions in the knee joint area”  “any adverse event (mild or moderate)”  “minor and transient adverse events”  “nervous system”  “non-serious adverse events”  “patients reporting adverse events”  “possibly drug related adverse event”  “possibly treatment related adverse events”  “severe adverse events”  “significant adverse events”  “systematic adverse effects possibly related to treatment”  “treatment-related adverse events”  aphtosis | **Serious**  cutaneous vasculitis  “skin reaction characterized by peeling of skin on hands and toes”  myocardial infarction  gastroduodenal ulcer  angina  gastrointestinal hemorrhage  gastrointestinal cancer  pneumonia  transient ischemic attack (TIA)  cancer (unknown type)  forearm fracture  intestinal obstruction  aggravated urinary incontinence  upper gastrointestinal bleeding  joint sprain  “potentially serious AE”  “treatment emergent adverse event”  “serious adverse event”  “SAE”  **Not serious**  flare  **Omitted from analysis in Rutjes et al. (due to lack of designation as serious by original study authors)**  myocardial infarction[52, 76]  severe knee swelling[76]  breast cancer[76] |
