# Supplementary material for: Hyaluronic acid injection therapy for osteoarthritis of the knee: concordant efficacy and conflicting serious adverse events in two systematic reviews
Source: Syst Rev. 2016 Nov 4;5:186. doi: 10.1186/s13643-016-0363-9 (PMC5097414; doi:10.1186/s13643-016-0363-9)
Supplement: Additional file 6: Table S6. — Comparison of AE and SAE categorization in our review and the review by Rutjes and colleagues [8]. Both articles categorized AEs as serious or non-serious. Our review also categorized by locality (joint, non-joint local, or other). Both reviews omitted some SAEs that were qualitatively similar to SAEs counted by the other group. Our review excluded events only that did not describe the type of SAE that occurred. Rutjes et al. omitted some events that were similar to events they did count as SAEs. (DOCX 38 kb) [file 13643_2016_363_MOESM6_ESM.docx]

**Appendix 6: Comparison of AE and SAE categorization in our review and the review by Rutjes and colleagues.[8]**

| **AEs included in AE and SAE analyses in our review** | **AEs included in AE and SAE analyses by Rutjes et al.** |
| --- | --- |
| **Serious, joint**  acute synovitis  flare at the knee joint  haemarthrosis  post-injection flares  severe knee swelling and/or effusion  **Serious, local**  [none]  **Serious, other**  influenza-like symptoms  skin disorders  death  fever  forearm fracture  intestinal obstruction  myocardial infarction  skin reaction (peeling on hands and toes, erythema)  upper gastrointestinal bleeding  **Not serious, joint**  knee pain during or after injection  arthralgia  effusion  increase in effusion volume  knee pain after injection related to the injection procedure  local joint pain and swelling  musculoskeletal  stiffness in the index knee  transient increase in pain/swelling in the treated knee  **Not serious, local**  cutaneous vasculitis  erythema at the injection site  injection site pain  injection site reactions  local reaction at the injection site with pain, tenderness and erythema  local reactions at the injection site  local skin (ecchymosis and rash)  minor discomfort during the injection  pain and local swelling at the injection site  pain and swelling  painful injection  pruritis (local)  skin  **Not serious, other**  central and peripheral nervous disorders (headache, vertigo)  diarrhea  gastralgia  nausea, vomiting  other gastrointestinal disorders  respiratory system disorders (bronchitis, rhinitis…)  urine coloration  aggravated urinary incontinence  gastrointestinal  gastrointestinal complaints  general body  headache  joint sprain  phlebitis  pruritus  **Omitted from our analysis (due to lack of specificity in AE or SAE description in original article)**  “adverse events not associated with local reactions in the knee joint area”  “any adverse event (mild or moderate)”  “minor and transient adverse events”  “nervous system”  “non-serious adverse events”  “patients reporting adverse events”  “possibly drug related adverse event”  “possibly treatment related adverse events”  “severe adverse events”  “significant adverse events”  “systematic adverse effects possibly related to treatment”  “treatment-related adverse events”  aphtosis | **Serious**  cutaneous vasculitis  “skin reaction characterized by peeling of skin on hands and toes”  myocardial infarction  gastroduodenal ulcer  angina  gastrointestinal hemorrhage  gastrointestinal cancer  pneumonia  transient ischemic attack (TIA)  cancer (unknown type)  forearm fracture  intestinal obstruction  aggravated urinary incontinence  upper gastrointestinal bleeding  joint sprain  “potentially serious AE”  “treatment emergent adverse event”  “serious adverse event”  “SAE”  **Not serious**  flare  **Omitted from analysis in Rutjes et al. (due to lack of designation as serious by original study authors)**  myocardial infarction[52, 76]  severe knee swelling[76]  breast cancer[76] |

Both articles categorized AEs as serious or non-serious. Our review also categorized by locality (joint, non-joint local, or other). Both reviews omitted some SAEs that were qualitatively similar to SAEs counted by the other group. Our review excluded events only that did not describe the type of SAE that occurred. Rutjes et al. omitted some events that were similar to events they did count as SAEs.
